# Human precursor T follicular regulatory cells are primed for differentiation into mature Tfr and disrupted during severe infections

**DOI:** 10.1126/sciadv.adv6939

**Published:** 2025-09-26

**Authors:** Janyerkye Tulyeu, Jonas N. Søndergaard, David G. Priest, Takeshi Ebihara, Hisatake Matsumoto, Mara A. Llamas-Covarrubias, Masaki Imai, Shinichi Esaki, Shinichi Iwasaki, Akimichi Morita, Sayuri Yamazaki, Shimon Sakaguchi, James B. Wing

**Affiliations:** ^1^ Human Single Cell Immunology Team, CiDER, The University of Osaka, Osaka, Japan.; ^2^ Laboratory of Human Single Cell Immunology, IFReC, The University of Osaka, Osaka, Japan.; ^3^ Department of Traumatology and Acute Critical Medicine, The University of Osaka Graduate School of Medicine, Osaka, Japan.; ^4^ Department of Immunology, Nagoya City University Graduate School of Medical Sciences, Nagoya, Japan.; ^5^ Department of Otorhinolaryngology, Head and Neck Surgery, Nagoya City University Graduate School of Medical Sciences, Nagoya, Japan.; ^6^ Department of Geriatric and Environmental Dermatology, Nagoya City University Graduate School of Medical Sciences, Nagoya, Japan.; ^7^ Laboratory of Experimental Immunology IFReC, The University of Osaka, Osaka, Japan.; ^8^ Department of Experimental Pathology, Institute for Frontier Medical Sciences, Kyoto University, Kyoto, Japan.; ^9^ Center for Advanced Modalities and DDS (CAMaD), The University of Osaka, Osaka, Japan.

## Abstract

T follicular regulatory cells (Tfr) play a pivotal role in maintaining immune self-tolerance and prevention of autoantibody induction. However, the stages of Tfr formation have still not been clarified. Here, we found that 30 to 50% of circulating Tfr in human blood have a naïve-like phenotype (preTfr, CD45RA^+^CD45RO^−^Foxp3^+^CXCR5^+^). PreTfr are expandable in vitro while retaining suppressive capacity. They are transcriptionally similar to naïve T_regs_ (nT_regs_) ex vivo, but after stimulation, they gain increased expression of Tfr-related suppressive molecules such as IL-1RA, suggesting that they are primed for full differentiation into mature Tfr. Further, we demonstrate that preTfr but not nT_reg_ are significantly reduced in the blood of patients with COVID-19 and patients with sepsis and that this loss is correlated with increased anti–interferon-γ antibodies and activated atypical B cells. Together, these data suggest that Tfr are disrupted at the earliest stage of their formation during severe disease and may be an important therapeutic target in future vaccine developments.

## Teaser

The loss of naïve-like precursor T follicular regulatory cells during COVID-19 correlates with autoantibody production.

## INTRODUCTION

Regulatory T cells (T_regs_), characterized by the expression of the transcription factor Foxp3, play an important role in maintaining immune homeostasis, including the regulation of humoral immunity (*1*–*3*). T_regs_ exhibit substantial phenotypic and functional heterogeneity, with recent studies shedding light on distinct subsets within this population (*4*–*6*). T follicular regulatory (Tfr) cells are a T_reg_ subset specialized in modulating immune responses within germinal centers (GCs) by controlling T follicular helper (Tfh) cell–driven GC responses (*7*, *8*). By suppressing Tfh cells, which promote B cell maturation and antibody production, they help to maintain immune homeostasis and prevent autoantibody generation (*8*).

Dysregulation of the Tfr/Tfh cell ratio has been implicated in various autoimmune conditions such as rheumatoid arthritis and systemic lupus erythematosus, suggesting that an imbalance between these two cell types can drive pathogenesis (*9*–*11*). Moreover, Tfr cells are increasingly being studied for their role in vaccine responses, where their ability to modulate immune tolerance and antibody production could provide insights into optimizing immunization strategies (*12*–*14*). In recent years, Tfr cells have also been explored in the context of viral infections, including COVID-19 (*15*). In severe COVID-19 cases, altered Tfr cell functionality has been associated with dysregulated immune responses, excessive antibody production, autoantibody production, and inflammation (*16*). This dysfunction may hinder the suppression of hyperactive immune responses, contributing to cytokine storms and severe complications (*17*, *18*). The differentiation of Tfr cells in these settings is influenced by cytokines such as interleukin-12 (IL-2) and IL-12, which also modulate the activity of Tfh (*19*). Despite the important role of Tfr in diverse immunological conditions, the exact underlying mechanism of Tfr formation and function still has not been clarified (*8*).

Previously, we and others have demonstrated that Tfr have a high frequency in human blood and lymphoid organs, making up as many as 50% of total T_regs_ in sites such as the tonsils (*7*, *20*–*22*). A few groups have reported the presence of CD45RA^+/−^ Tfr cells but have not investigated the differences (*13*, *22*). It has been hypothesized that the CD45RA^+^ subset is a progenitor to the more active CD45RA^−^ subset (*13*, *22*), although no direct evidence for this hypothesis has been presented. Understanding the phenotypic and functional nuances of Tfr cells, particularly the circulating naïve-like CD45RA^+^ Tfr subset (preTfr), is critical for advancing our knowledge of immune regulation and developing therapeutic strategies for autoimmune diseases and other immune-related conditions. Here, we elucidated the characteristics, functions, and clinical relevance of preTfr cells in both healthy and diseased states, providing insights into their roles in immune homeostasis and pathology.

## RESULTS

### Identification of CD45RA-expressing Tfr in blood

**Fig. 1.**
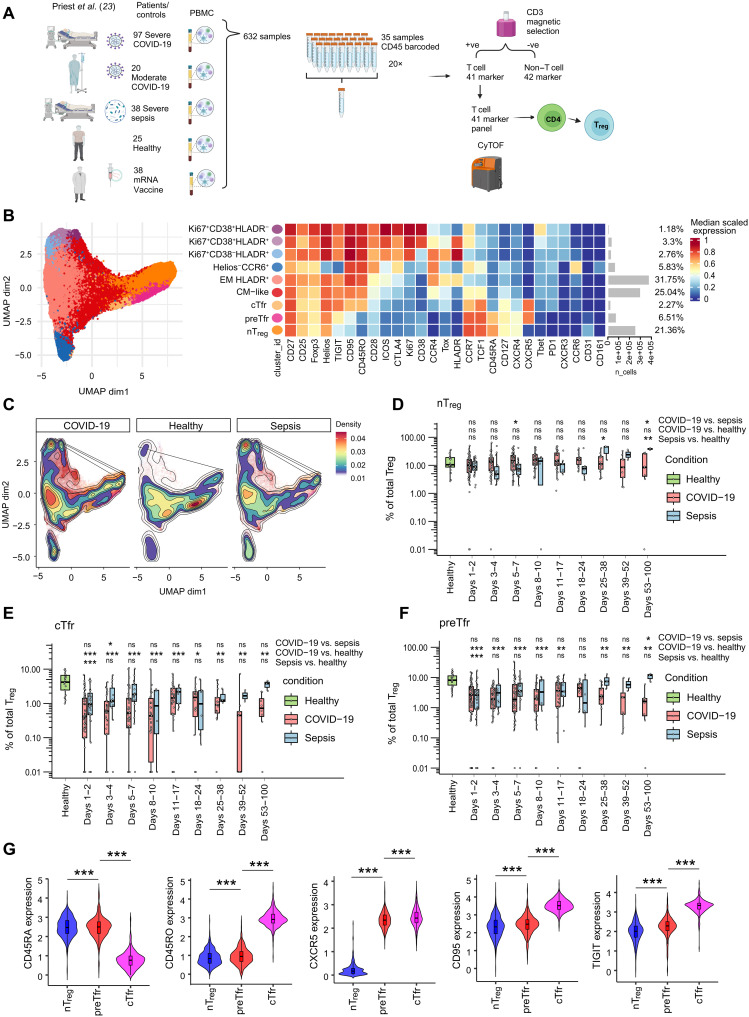
Mass cytometry analysis of T_regs_ reveals distinct subpopulations and alterations in patients with COVID-19 and patients with sepsis. (**A**) Schematic representation of the patient cohort and study design. Created in BioRender. Wing, J. (2025) https://BioRender.com/qybop5w. (**B**) UMAP visualization and heatmap illustrating the median scaled expression markers of T_reg_ FlowSOM clusters. (**C**) Density UMAP of T_reg_ subsets split by condition. UMAP, Uniform Manifold Approximation and Projection. (**D** to **F**) Proportion of T_reg_ subsets in patients with COVID-19 and patients with sepsis across days since admission to ICU compared to age-matched healthy controls over time. Statistical significance [false discovery rate (FDR)–adjusted *P* values from edgeR] is indicated with *P < 0.05, **P < 0.01, and ***P < 0.001. ns, nonsignificant. The other T_reg_ populations are presented in fig. S1. Healthy, *n* = 25. Days 1 and 2, COVID-19 *n* = 85, sepsis *n* = 34; days 3 and 4, COVID-19 *n* = 43, sepsis *n* = 14; day 5 to 7, COVID-19 *n* = 52, sepsis *n* = 20; day 8 to 10, COVID-19 *n* = 38, sepsis *n* = 4; days 11 to 17, COVID-19 *n* = 38, sepsis *n* = 9; days 18 to 24, COVID-19 *n* = 16, sepsis *n* = 6; days 25 to 38, COVID-19 *n* = 17, sepsis *n* = 3; day 39 to 52, COVID-19 *n* = 9, sepsis *n* = 2; day 53 to 100, COVID-19 *n* = 8, sepsis *n* = 3. (**G**) Violin plots of protein expression in T_reg_ clusters pooled from all time points of severe COVID-19, severe sepsis, and healthy. Statistical significance (two-sided Wilcoxon rank sum test corrected for donor differences and Benjamini-Hochberg adjusted *P* values, *n* = 424) is indicated with ***P < 0.001. For all boxplots, center line is median, hinges correspond to the first and third quartiles, and whiskers correspond to the 1.5 times interquartile range. CM, central memory.

To perform an in-depth examination of T_regs_ and Tfr in humans, we reanalyzed our previously published human peripheral blood mononuclear cell (PBMC) dataset examining a large longitudinal cohort of patients with severe COVID-19 and patients with bacterial sepsis alongside a health care worker cohort of Pfizer mRNA vaccine recipients (*23*) to focus on T_reg_ subset phenotypes (Fig. 1, A and B). All samples were analyzed in a single combined workflow allowing in-depth comparison of cellular states across our separate cohorts (*23*). Analysis of total lineage populations in our intensive care unit (ICU) cohort showed that the frequency of total T_reg_ as a proportion of PBMC was not changed at any time point in COVID-19 and sepsis (fig. S1, A to C). We then performed a detailed analysis of T_regs_ and annotated the clusters based on our previous work and recent studies (*15*, *24*). We identified a variety of T_reg_ subsets including proliferating CD38^hi^ and/or HLA-DR^hi^ T_reg_ groups that we have described previously (*15*, *25*). In the context of Tfr, in addition to the well-known CD45RO^+^ effector Tfr (eTfr), we were also able to observe a group of CD45RA^+^CXCR5^+^ cells that we here refer to as naïve-like precursor Tfr (preTfr) cells (Fig. 1B).

### preTfr frequency is altered in patients with COVID-19 and patients with sepsis

We then examined changes to the proportions of T_regs_ in our longitudinal ICU cohort (Fig. 1, C to F, and fig. S1, D to I). We found that both circulating effector Tfr (cTfr) and preTfr cells were significantly reduced at all measured time points in COVID-19 and early stages in sepsis in comparison to age-matched healthy controls (Fig. 1, E and F). In contrast, naïve T_regs_ (nT_regs_) were largely stable during COVID-19 and sepsis, indicating that preTfr and nT_reg_ have different disease response patterns (Fig. 1D). Principal components analysis (PCA) of healthy controls showed that preTfr were more similar to nT_regs_ than cTfr (fig. S2D). Phenotypically, the preTfr was primarily separated from nT_regs_ by CXCR5, although we also found TIGIT, CD45RO, and CD95 to be slightly higher in preTfr, with CD45RA and CD31 lower (Fig. 1G and fig. S2B). However, while the differences in CD45RA, CD45RO, and CD95 were statistically significant, indicating a consistent difference in this large dataset, they were also extremely small. As a result, the population was only truly separated by CXCR5 allowing us to broadly characterize the preTfr as naïve like.

Comparing preTfr to cTfr showed large differences in classic maturation markers such as CD45RO and CD95 being higher on cTfr and CD45RA, TCF1 (TCF7), CD31, and CCR7 being higher on preTfr (Fig. 1G and fig. S2B). In COVID-19 and sepsis, we found similar results regarding marker expression as for the healthy controls (fig. S2, C and D). cTfr, preTfr, and nT_reg_ populations all had higher expression levels of CD95 and CXCR4 in COVID-19 and sepsis compared to healthy controls (fig. S3, A to C). Widespread up-regulation of CXCR4 in T cells appears to be a common feature of severe infection as we described previously (*15*). We next examined associations with sex and mortality. As previously reported (*15*), we found that Tfr trended toward being reduced in male patients compared to female (fig. S4, A to C). Kaplan-Meier analysis showed that mortality in COVID-19 was affected by the frequency of preTfr (*P* = 0.052), with a lower preTfr frequency resulting in increased mortality (fig. S4B). In contrast, cTfr frequency did not affect mortality, and nT_reg_ had a similar trend as preTfr (*P* = 0.150). These data suggest that preTfr may have a protective function in severe COVID-19.

### preTfr frequency negatively correlates with anti-cytokine autoantibodies

**Fig. 2.**
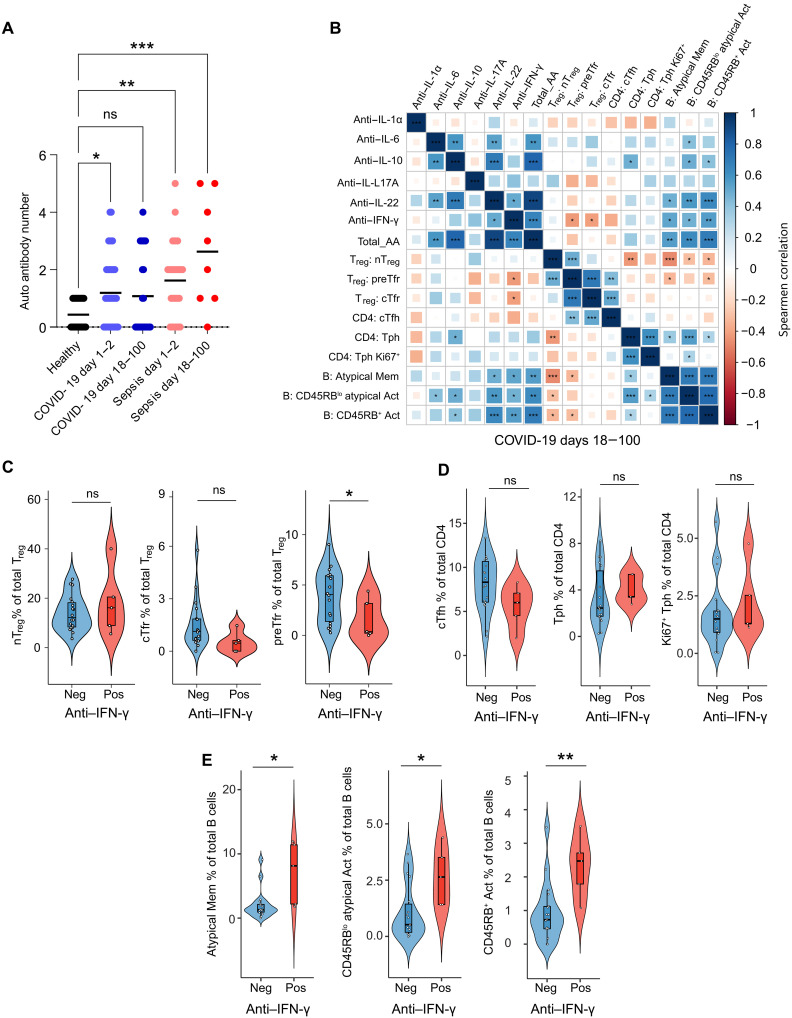
Negative correlation between preTfr cells and anti-cytokine autoantibody levels. (**A**) Dot plot shows the total number of autoantibodies in COVID-19 and sepsis compared to healthy controls (healthy *n* = 23, COVID-19 *n* = 89, sepsis *n* = 36). Significance *P < 0.05, **P < 0.01, and ***P < 0.001 by repeated measures one-way analysis of variance (ANOVA) and Holm-Sidak posttest. (**B**) Spearman rank correlation matrix of indicated autoantibodies, B cell (atypical memory, CD45RB^lo^ activated atypical, and CD45RB^+^ activated), CD4 helper (cTfh and Tph), and T_reg_ subsets in late COVID-19 (days 18 to 100, healthy *n* = 23, COVID-19 *n* = 89, sepsis *n* = 36). Significance *P < 0.05, **P < 0.01, and ***P < 0.001 by two-sided Spearman’s rank correlation. (**C** to **E**) Violin plots depicting the frequency of indicated T_reg_, CD4 helper, and B cell subsets, in patients with COVID-19 grouped by detection of anti–IFN-γ autoantibody (positive: MFI measurement greater than 2 SD above the average MFI for healthy controls). Statistical significance is derived from unpaired Wilcoxon sign rank tests *P < 0.05, healthy *n* = 23, COVID-19 *n* = 89, sepsis *n* = 36. Hinges in boxplots correspond to the first and third quartiles, center line is median, and whiskers correspond to the 1.5 times interquartile range.

Tfr have a key role in the regulation of autoantibodies (*17*), and anti-cytokine autoantibodies have been demonstrated to be induced during COVID-19 (*26*). To explore the potential regulatory role of preTfr cells in autoantibody production, we measured the plasma autoantibody levels in our patient cohort and compared it to the T_reg_ subset frequencies (Fig. 2 and fig. S5). The total number of autoantibodies was higher in COVID-19 and sepsis compared to healthy controls (Fig. 2A and fig. S5A). Of seven measured anti-cytokine autoantibodies, we saw significant correlations between the preTfr and cTfr subsets and the anti–interferon-γ (IFN-γ) autoantibodies in late COVID-19 (days 18 to 100; Fig. 2B) but not in late sepsis (fig. S5B) or early COVID-19 (days 1 to 2; fig. S5C). The nT_reg_ response was more dynamic, having a slightly negative correlation with anti–IFN-γ autoantibodies only in early COVID-19 (fig. S5C) and a negative correlation with anti–IL-10 autoantibodies only in early sepsis (fig. S5D). However, the overall nT_reg_ frequency was higher in patients with anti–IFN-γ autoantibody–positive COVID-19, while both circulating T follicular helper (cTfr) and preTfr percentages were lower (Fig. 2C). The cTfh and T peripheral helper (Tph) subsets lacked a significant difference in the same analysis (Fig. 2D) Furthermore, in late COVID-19, the atypical, CD45RB^lo^ atypical activated (CD45RB^lo^ atypical act) and CD45RB^+^ activated B cell subsets that we previously identified (*23*), had a strong positive correlation with several anti-cytokine autoantibodies (IL-6, IL-10, IL-22, and IFN-γ) as well as Tph cells but a negative correlation with T_reg_ subsets including preTfr (Fig. 2B). These B cells were significantly higher in patients with anti–IFN-γ autoantibodies (Fig. 2E). Together, these data thus indicate that preTfr and cTfr cells may contribute to the regulation of autoantibody production in late COVID-19.

### preTfr respond to SARS-CoV-2 mRNA vaccination

**Fig. 3.**
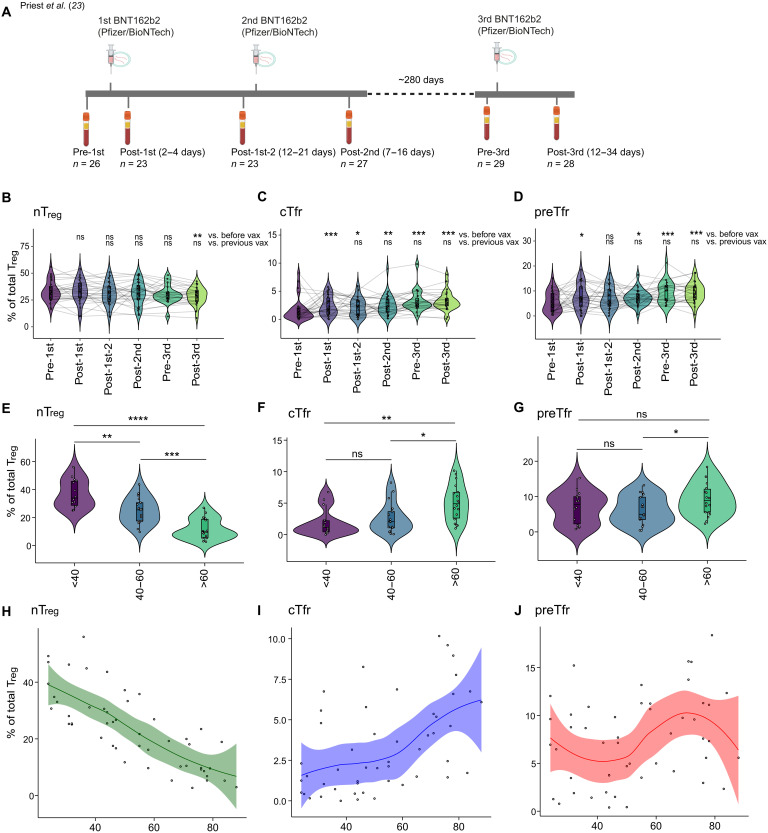
Response of preTfr cells to SARS-CoV-2 mRNA vaccination. (**A**) Schematic illustration of the SARS-CoV-2 mRNA vaccination cohort, including the time points at which samples were collected (*23*). Created in BioRender. Wing, J. (2025) https://BioRender.com/ypwuk86. (**B** to **D**) Proportions of T_reg_ clusters within the SARS-CoV-2 mRNA vaccination cohort over time. Statistical significance is FDR-adjusted *P* values derived from edgeR and represented with *P < 0.05, **P < 0.01, ***P < 0.001. Pre-first *n* = 28; post-first *n* = 26; post-first–2 *n* = 22; post-second *n* = 28; pre-third *n* = 19; post-third *n* = 17. (**E** to **G**) Violin plots of healthy donor T_reg_ frequency binned by age as <40 (*n* = 14), 40 to 60 (*n* = 17), and >60 (*n* = 18). Statistics: ANOVA with a Tukey posttest. (**H** to **J**) Dot plots of T_reg_ subset frequency versus age. For all boxplots, center line is median, hinges correspond to the first and third quartiles, and whiskers correspond to the 1.5 times interquartile range. vax, vaccination.

We next examined the response of nT_reg_ and preTfr cells to severe acute respiratory syndrome coronavirus 2 (SARS-CoV-2) mRNA vaccination in our longitudinal cohort of health care workers who received the BNT16b2 mRNA vaccine (Fig. 3A) (*23*). We found significant increases in both preTfr and cTfr frequency following vaccination, particularly at later time points, while nT_regs_ remained largely unchanged (Fig. 3, B to D). These findings suggest that although Tfr populations are rare, they increase in frequency to mRNA vaccination, potentially playing a role in the vaccine-induced immune response (*27*, *28*). In the same cohort, we previously demonstrated expansion of circulating Tfh (*23*) underlining that Tfh and Tfr expand at the same time, presumably due to reacting against the same stimuli. We then examined the relationship of Tfr frequency with neutralizing antibody levels and other vaccine-responsive subsets such as the B cell and CD4^+^ T cell subsets described in our previous analysis (*23*) and CD8^+^ T cell subsets examined in further detail here (fig. S6, A to C). Correlation analysis of the data aggregated across time points demonstrated that both preTfr and cTfr positively correlated with cTfh and the CD45RB^lo^ activated atypical B cells that we previously found to be strongly associated with anti–receptor-binding domain (RBD)-, anti–S1-, and anti–S2–immunoglobulin G (IgG) antibody levels (*23*) and here also found to be positively correlated with neutralizing antibodies (fig. S6D). However, only cTfr but not preTfr showed a statistically significant positive correlation with neutralizing antibodies (fig. S6D). In contrast, nT_regs_ were either not correlated or negatively correlated with the expansion of vaccine reactive subsets and antibody levels. CD8^+^ T cell Ki67^+^ effector memory (EM) and T effector memory cells re-expressing CD45RA (TEMRA) subsets responded to the vaccination (fig. S6B) but did not show any correlation with preTfr or cTfr (fig. S6, D and E) likely indicating that these cells are not directly interacting in the same way as the Tfr do with Tfh and B cells. While correlation across time point indicates a broad pattern of expansion following vaccination, close examination of correlations restricted to the peak of the antibody response (post-third vaccination) was less clear (fig. S6E). The relationship between cTfr, cTfh, and CD45RB^lo^ activated atypical B cells was still significant, but the preTfr had weaker correlations with these subsets at the single time point. We next examined whether nT_regs_, cTfr, and preTfr had associations with age in healthy donors in our cohort (Fig. 3, E to J). While nT_regs_ were reduced with age and cTfr increased, preTfr did not have a clear relationship with age (Fig. 3, E to J). This suggests that preTfr may be a more stable subset over time compared to other T_reg_ subsets.

### Naïve-like Tfr have a distinct transcriptional profile

**Fig. 4.**
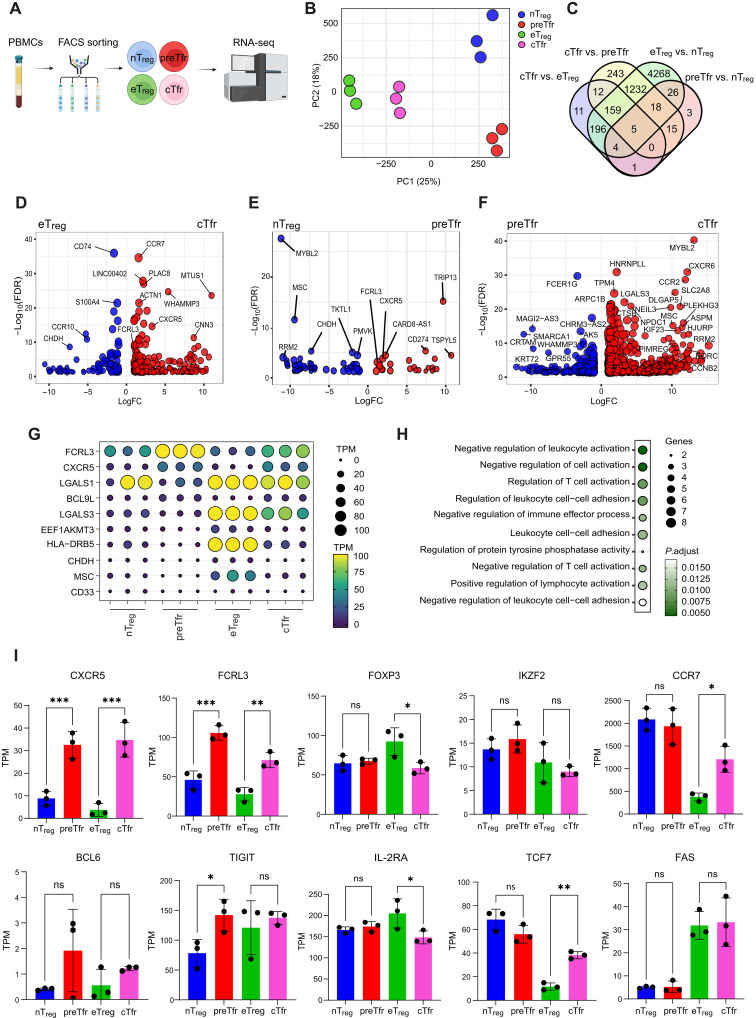
RNA-seq analysis of naïve and effector T_regs_ and Tfr cells. (**A**) Schematic representation of the RNA-seq experiment workflow. Created in BioRender. Wing, J. (2025) https://BioRender.com/yqk8hhn. (**B**) PCA plot visualizing the gene expression profiles of naïve and effector T_regs_ and Tfr cells. (**C**) Venn diagram illustrating the overlap of differentially expressed genes (DEGs) identified between effector T_reg_ versus naïve T_reg_ and circulating Tfr versus precursor Tfr comparisons. (**D**) Volcano plot depicting DEGs between effector T_reg_ (blue) and Tfr (red) populations. (**E**) Volcano plot showing DEGs between naïve T_reg_ (blue) versus Tfr (red) populations. (**F**) Volcano plot showing DEGs between preTfr (blue) versus circulating Tfr (red) populations. FC, fold change. (**G**) Dot plot displaying the expression levels of 10 common DEGs across all comparisons. (**H**) Dot plot summarizing the results of gene ontology enrichment analysis for the identified DEGs. (**I**) mRNA expression levels of selected genes of interest. Statistics: [(D) to (F) and (I)] edgeR corrected for donor differences, *n* = 3 per condition, *P < 0.05, **P < 0.01, ***P < 0.001.

Next, we sought to determine how preTfr differed from nT_regs_ as our initial mass cytometry (CyTOF) panel only identified CXCR5 and TIGIT as strongly distinguishing markers (fig. S2, B to D). Thus, we performed RNA sequencing (RNA-seq) analysis to examine the transcription-wide profiles, providing a molecular basis for understanding their functional differences. To this end, preTfr, cTfr, nT_reg_, and effector (e)T_regs_ were isolated from three healthy donor PBMCs by fluorescence-activated cell sorting (FACS), and purified cell populations were analyzed by RNA-seq (Fig. 4A and fig. S7). PCA of the gene expression data revealed a clear separation between the naive and effector T_reg_ and Tfr populations, reflecting distinct transcriptional landscapes (Fig. 4B). To identify key transcriptional differences, we performed differential expression analysis, comparing effector T_reg_ to Tfr populations and naive T_reg_ to Tfr populations (Fig. 4, C to F). By comparing the overlap of these (Fig. 4C), 10 genes were consistently different between T_reg_ and Tfr subsets irrespective of whether naïve or effector subtypes, including CXCR5 and the Tfr-associated repressive molecule Fc receptor-like protein 3 (FCRL3) (Fig. 4G) (*29*). Gene ontology analysis revealed that these genes were involved in negative regulation of leukocyte activation and adhesion (Fig. 4H). Concerning additional classic T_reg_ and Tfr-related markers, we did not observe any significant differences in expression of FOXP3, IKZF2 (Helios), IL-2RA, or TCF7 between nT_reg_ and preTfr, but TIGIT was significantly higher in preTfr, and BCL6 trended toward being higher in preTfr but was essentially very low (Fig. 4I). In keeping with previous reports, compared to eT_regs_, cTfr had a reduced level of IL-2RA (*20*) and increased TCF7 (*30*). Furthermore, higher expression of CCR7 and lower expression of FAS of both nT_regs_ and preTfr compared to the effector subsets indicate a naïve cellular phenotype. While it has been previously suggested that circulating Tfr as a whole (i.e., preTfr and cTfr together) have a less developed phenotype (*13*), our results suggest that while they share some core transcriptional signature, cTfr and preTfr are otherwise more similar to eT_regs_ and nT_regs_, respectively, than they are to one another, indicating that preTfr is a distinct subset with a specific transcriptional profile suggesting specific functional properties.

### preTfr are expandable and have suppressive activity in vitro

**Fig. 5.**
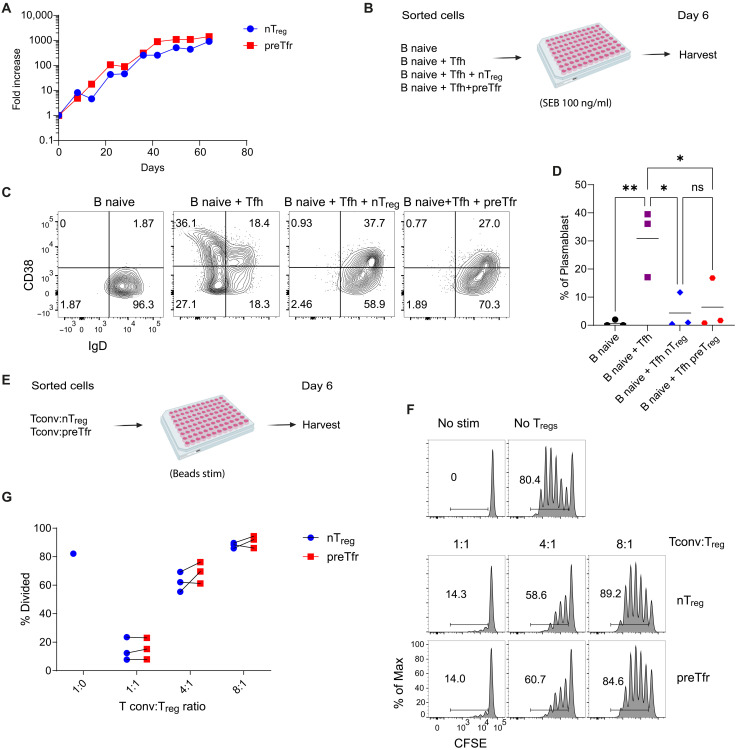
In vitro expansion and T_reg_ suppression assays. (**A**) Line graph depicting the expansion of nT_regs_ and Tfr cells over time (days in culture, *n* = 1). Another replicate can be seen in fig. S7A. (**B**) Schematic representation of the in vitro B cell suppression assay. (**C**) Gating strategy for identifying plasmablasts under different conditions. (**D**) Dot plots showing the percentage of plasmablasts in different conditions. Statistics: Repeated measures one-way ANOVA with a Holm-Sidak posttest, *n* = 3 per condition. *P < 0.05 and **P < 0.01. (**E**) Schematic representation of the in vitro T cell suppression assay. (**F**) Histograms comparing the percentage of proliferating Tconv across different conditions. (**G**) Percentage of proliferating T cells in the presence of varying ratios of T_regs_. *n* = 3. Tconv, T conventional. [(B) and (E)] Created in BioRender. Wing, J. (2025) https://BioRender.com/nwkkdlz.

To directly assess the functional capacity of preTfr, we performed in vitro expansion and suppression assays (Fig. 5). nT_reg_ and preTfr was purified by FACS sorting from human PBMCs and stimulated with IL-2, IL-12, and activin A for up to 64 days. Both preTfr and nT_reg_ were equally expandable for this extended period (Fig. 5A and fig. S8A), indicating that they both are naïve cells with stem-like expandability. Next, we used a B cell suppression assay to evaluate the suppressive effects of preTfr on B cell activation (Fig. 5B). By measuring the amount of plasmablasts generated from naïve B cells after 6 days in culture, we saw that both nT_reg_ and preTfr were equally good at suppressing plasmablast generation (Fig. 5, C and D). Similarly, both preTfr and nT_reg_ were able to suppress conventional T cell proliferation in a classic T_reg_ suppression assay in both a bead-based (Fig. 5, E to G) and antigen-presenting cell (APC)-based assay format (fig. S8, B and C). Together, these data suggest that preTfr are functionally similar to nT_regs_ in terms of immune suppression and expandability.

### preTfr secrete IL-1RA, which suppresses plasmablast generation

**Fig. 6.**
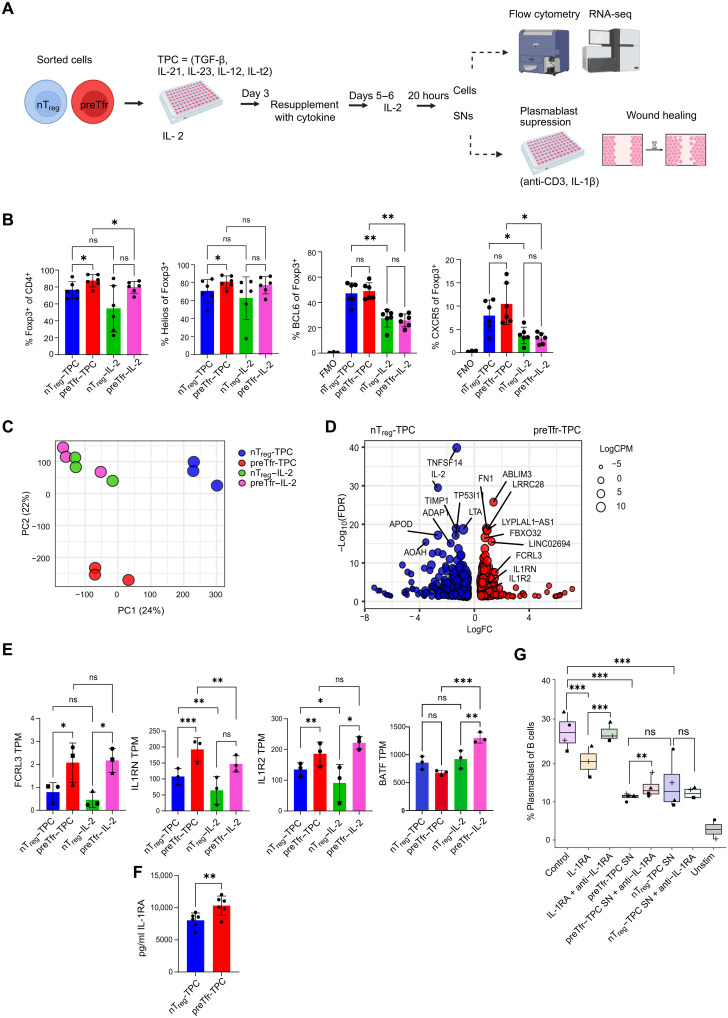
Precursor Tfr secrete IL-1RA, which suppresses plasmablast generation. (**A**) Schematic diagram outlining the study design for TPC. Created in BioRender. Wing, J. (2025) https://BioRender.com/qx390a1. TGF-β, transforming growth factor–β. (**B**) Flow cytometry analysis showing the levels of BCL6 and CXCR5 expression in T_regs_ from different groups. Statistics: Repeated measures one-way ANOVA with a Holm-Sidak posttest, *n* = 6 (FMO only, *n* = 3). (**C**) PCA plot visualizing the gene expression profiles of TPC- and IL-2–treated T_regs_ and Tfr cells. (**D**) Volcano plot depicting DEGs between nT_reg_-TPC (blue) and preTfr-TPC (red) populations. Statistics: edgeR corrected for donor differences, *n* = 3 per condition. (**E**) mRNA expression level of selected genes. Statistical significance (adjusted *P* values from edgeR) is indicated with *P < 0.05, **P < 0.01, and ***P < 0.001. (**F**) Boxplot showing protein expression level of ILRA determined by ELISA of T_reg_ subset SNs. Statistics: Two-tailed paired *t* test, *n* = 6. (**G**) Boxplots of plasmablast suppression assay in total PBMCs treated with TPC culture SNs. Statistics: edgeR corrected for donor differences, *n* = 3 to 4 per condition (unstim, *n* = 2 but not used for statistical comparison). Hinges correspond to the first and third quartiles, center line is median, and whiskers correspond to the 1.5 times interquartile range. FMO, flourescence minus one; unstim, unstimulated.

To further address the stability and function of preTfr after activation, we used conditions previously shown to promote Tfh-like characteristics in T cells, such as BCL6 expression in T_regs_ (*31*, *32*) with some modifications, here referred to as Tfr-promoting conditions (TPC) (Fig. 6A). We first assessed the phenotype of nT_reg_ and preTfr cultured under these conditions by flow cytometry and compared the results to cells cultured using IL-2 alone (Fig. 6B and fig. S9, A and B). Under TPC, preTfr had slightly higher Foxp3 and Helios expression than nT_regs_ (Fig. 6B). TPC conditions were also able to induce/maintain BCL6 and CXCR5 expression in both nT_reg_ and preTfr but without a clear difference between the subsets. To gain further insights into transcriptional differences between nT_reg_ and preTfr cultured under TPC or IL-2 conditions, we used RNA-seq (Fig. 6, C to E). PCA analysis showed that under IL-2 conditions, there were few differences between nT_reg_ and preTfr, but under TPC conditions, they strongly diverged (Fig. 6C). The suppressive molecules FCRL3, IL1RN (encoding the IL-1RA protein), and IL1R2 were all higher in preTfr regardless of conditions (Fig. 6, D and E). While FCRL3 was different even prestimulation (Fig. 4I), IL1RN and IL1R2 were not detectable in ex vivo cultured preTfr (fig. S8, C and D). We further validated the higher level of secreted IL-1RA by preTfr at the protein level by enzyme-linked immunosorbent assay (ELISA) (Fig. 6F). Since IL-1RA is a Tfr-associated molecule known to inhibit antibody production (*33*), we investigated whether the additional secreted IL-1RA influenced plasmablast formation (Fig. 6G). We used preTfr and nT_reg_-derived supernatants (SNs) in a T cell–driven total PBMC suppression assay using CyTOF (Fig. 6G and fig. S10, A to F). As controls, we demonstrated that recombinant IL-1RA reduced plasmablast formation and that this could be rescued by anti–IL-1RA blocking monoclonal antibodies (Fig. 6G). Both preTfr and T_reg_ SNs inhibited plasmablast formation, but only the preTfr SN was significantly affected by anti–IL-1RA treatment. Furthermore, cTfh was inhibited by both preTfr and nT_reg_ SNs, but they were not responsive to the addition of IL-1RA or its blockade, while Tfr in culture itself were more sensitive to IL-1RA (fig. S10, D to F). These data suggest that IL-1RA is one of the effector molecules of preTfr that distinguishes them from nT_regs_.

### preTfr has enhanced wound healing capacity

**Fig. 7.**
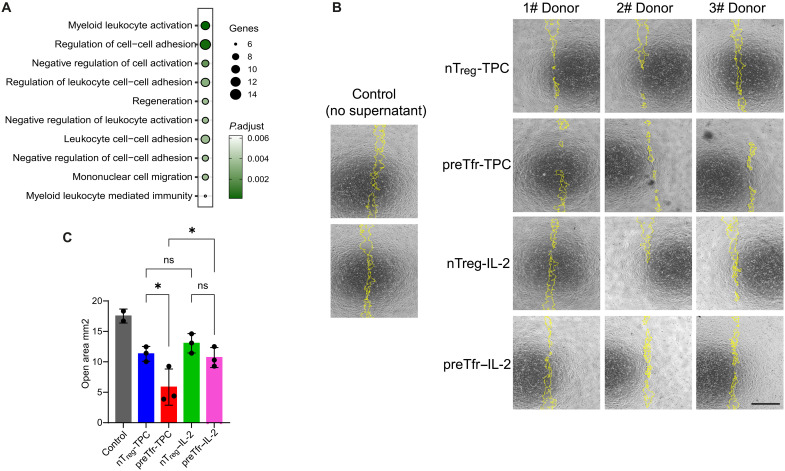
Precursor Tfr have increased wound healing capacity. (**A**) Dot plot summarizing the results of gene ontology enrichment analysis for genes differentially expressed between preTfr to nT_regs_ (adjusted *P* < 0.05 from edgeR corrected for donor differences, *n* = 3 per condition). (**B** and **C**) Microscopy images and summarizing bar plots of wound healing assay results. SNs from TPC or IL-2 groups of T_regs_ and Tfr cells from three donors were used in a wound healing assay with HaCaT cells (1:7 dilution, *n* = 3, yellow = open area analyzed with repeated measures one-way ANOVA and Holm-Sidak posttest, *P < 0.05). Scale bar, 4 μm. Controls (*n* = 2) are HaCaT cells with no T_reg_/Tfr SNs added.

A previous report demonstrated the T_regs_ subjected to Tfr-inducing conditions gained some wound healing capacity, suggesting that this is an additional characteristic of Tfr-like T_regs_ (*31*). Furthermore, gene ontology analysis of genes differentially expressed by preTfr under TPC conditions showed an effect on regeneration (Fig. 7A). Thus, we investigated the wound healing capacity of preTfr and nT_reg_ SNs (Fig. 7, B and C). To this end, human keratinocyte (HaCaT) cells were assayed for the ability to close a defined gap (cell-free area) in culture after treatment with or without preTfr and nT_reg_ SNs. The preTfr-TPC SN increased the wound healing capacity compared to nT_reg_-TPC or preTfr and nT_reg_ IL-2 SNs (Fig. 7, B and C). A previous study reported that tissue repair T_reg_ signature is associated with basic leucine zipper transcriptional factor ATF-like (BATF) expression (*31*). However, here, we only found BATF expression raised in preTfr IL-2 condition rather than the TPC condition and thus not clearly associated with the enhanced wound healing observed for the Tfr-TPC group (Fig. 6E). These data suggest a noncanonical function of preTfr and further distinguish them functionally from nT_regs_.

### preTfr and intermediate groups identified in human tonsils

**Fig. 8.**
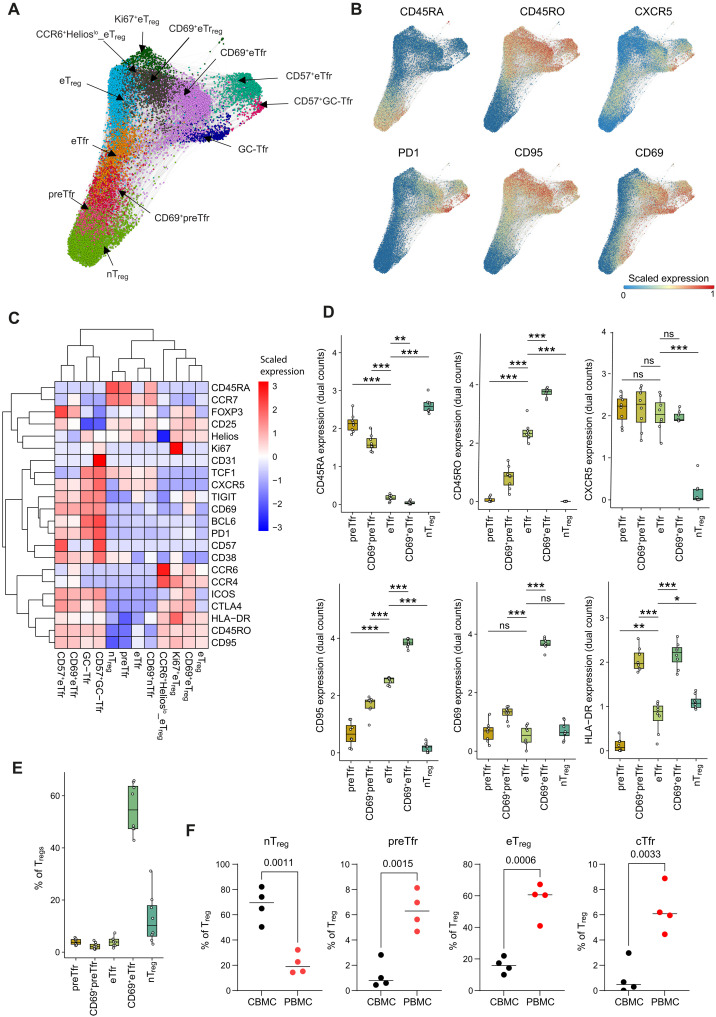
Mass cytometry analysis of T_reg_ populations in human tonsils. (**A**) ForceAtlas2 visualization of X-shift–clustered human tonsil T_regs_ (hypertrophy, *n* = 3 and IgA nephropathy, *n* = 5). (**B**) ForceAtlas2 feature plots showing the relative expression levels of indicated protein markers. (**C**) Heatmap illustrating the median scaled expression of type markers across merged T_reg_ clusters. (**D**) Boxplots of indicated protein relative expression levels from different cell types. (**E**) Boxplot of the frequency of selected T_reg_ clusters. (**F**) Dot plot shows a comparison between cord blood mononuclear cells (CBMC) and PMBC for different T_reg_ subsets. Statistics: Paired *t* test, *P* values indicated with *P < 0.05, **P < 0.01, and ***P < 0.001. Tonsil *n* = 8, cord blood *n* = 4, PBMC *n* = 4. For all boxplots, hinges correspond to the first and third quartiles, and whiskers correspond to the 1.5 times interquartile range.

While CXCR5-expressing circulating Tfr can be found in human blood, it remains essential to confirm their location and phenotype in key sites of their action such as human tonsils. To get a comprehensive picture of all T_reg_ subsets in human tonsil tissue, we used mass cytometry on a mixed cohort of tonsils derived from patients with hypertrophy and patients with IgA nephropathy (Fig. 8). Using X-shift–clustered data by ForceAtlas2, we were able to identify classic T_reg_ subsets such as nT_regs_, eT_regs_, and several groups of eTfr, notably including groups of cells expressing the tissue residency marker CD69, the CD25^lo^BCL6^+^PD1^+^ Tfr that we and others observed in previous work (*20*, *33*), and rare CD57^hi^ Tfr described in previous work (*34*) (Fig. 8, A to E, and fig. S11). Expression of CD38 may also indicate that some of these cells may have converted from non-T_regs_ (Tfh) (*35*). We also observed two groups of Tfr with high CD45RA expression, in close vicinity to the nT_regs_ on the force-directed layout (Fig. 8, A to C). These CD45RA-expressing Tfr (which we again annotated preTfr) also had other markers resembling naïve cells such as CCR7 and TCF1 while lacking effector markers such as CD45RO, PD1, and CD95 (Fig. 8, B to D, and fig. S11B). A subgroup of CD45RA^+^ Tfr also expressed a low level of CD69, CD45RO, and CD95 but was broadly speaking still naïve cells when compared to the eTfr groups. Since these cells express not only increased CD69 but also high levels of CD45RA and CCR7, it seems likely that these cells are in the early stages of transition from preTfr to the tissue resident CD69^+^ Tfr groups (Fig. 8, D and E, and fig. S11, C and D). Overall, these data suggest that preTfr are present in the tonsil and that CD69-expressing preTfr are at an early stage of transition to a true tissue resident Tfr phenotype. We additionally examined cord blood and compared the results to PBMCs from healthy individuals (Fig. 8F and fig. S12). The CD45RA-expressing Tfr were largely absent in cord blood as previously reported (*13*, *22*), suggesting that these cells develop in the periphery.

Together, we found that blood circulating preTfr have a transcriptional signature that is initially subtly different from nT_reg_, but on restimulation, they up-regulate Tfr suppression molecules such as IL-1RA and gain wound healing characteristics. While nT_regs_ are relatively unaffected by severe infections, preTfr in the blood are reduced during COVID-19 and correlate with increased levels of anti–IFN-γ autoantibodies. preTfr may thus be considered a distinct immune subset, with implications for future vaccine development strategies and immunobiology of inflammatory and infectious diseases.

## DISCUSSION

T_regs_ are essential for maintaining immune self-tolerance and homeostasis, preventing autoimmune reactions by regulating the immune response (*3*). Among the T_reg_ subsets, Tfr have been identified as a critical player in regulating antibody production (*21*, *36*, *37*). Despite the established importance of Tfr cells, their developmental stages and functional dynamics remain to be fully elucidated. Previous reports have demonstrated that some circulating Tfr have CD45RA expression (*13*, *22*, *38*), but the meaning of this has been unclear. A recent paper showed that both CD45RA^+^ Tfr and CD45RA^−^ Tfr were absent in the blood of patients with IL-12 receptor deficiency, suggesting that both forms of Tfr are formed following similar signals (*19*). Yang *et al.* (*30*) further suggested that total murine Tfr occupy a middle plane of activation, absent in both the most resting phenotype of T_regs_ and the most highly activated cells. We speculate that this overall middle activation level may be due to the absence of CD45RA^+/−^ division as it is unclear in this case if the mouse markers normally used for a similar purpose (CD44 and CD62L) have the same distribution. In line with the results of Yang *et al.* (*30*), we saw that the Tfr in lymph nodes (LNs) and GCs were broadly speaking effector cells but lacked some of the markers associated with the most highly activated T_reg_ populations such as CD103 and B lymphocyte-induced maturation protein-1 (BLIMP-1) (*20*). Human circulating Tfr also have relatively weak suppressive function leading to the suggestion that they are not fully licensed (*13*). Building on these previous findings, we here show that like T_regs_, Tfr cells can be categorized into distinct subsets based on their maturation state: In the blood, there are two groups—preTfr and cTfr cells. However, in the tonsils, a fuller range of activation states can be seen, with CD69^lo^ preTfr and eTfr populations similar to that seen in blood and thus may have recently arrived from circulation. Alongside these are CD69^+^ preTfr which may be the early precursors of the more activated groups of CD69^+^ eTfr and the distinctive subsets of CD25^lo^BCL6^hi^ Tfr.

Our transcriptome-wide analysis placed preTfr closer to nT_reg_ than cTfr yet constituting its own cluster on the PCA, indicating a unique gene expression profile. nT_regs_ have stem-like properties and are highly expandable in culture (*33*, *39*), and here we also found that preTfr showed potential for in vitro expansion while maintaining their suppressive capabilities. Upon stimulation, these cells up-regulated the Tfr-related suppressive molecules IL1R2 and IL-1RA (*38*, *40*) suggesting their priming for full differentiation into more mature Tfr cells. Although the SNs of both preTfr and nT_reg_ were able to inhibit plasmablasts formation in vitro, only the preTfr SN was affected by blocking of IL-1RA with anti–IL-1RA antibodies. Of note, this was only a partial rescue, and secreted IL-1RA is likely to be only one part of the suppressive molecules secreted by these cells. In cell-based suppression, we did not observe any significant differences in the ability between preTfr or nT_reg_ to suppress T cell proliferation or Tfh-mediated plasmablast generation, with both being excellent suppressors. This may suggest that the location of the cells in vivo is more important than their suppressive molecule expression, with Tfr expressing the characteristic follicle-homing receptor CXCR5 (*8*, *41*). Although the overall suppression was not different, we did observe some inhibitory receptors being differentially expressed. As previously reported (*29*), FCRL3 was higher on Tfr than T_regs_. Here, we demonstrated that this is the case for both preTfr (compared to nT_reg_) and cTfr (compared to eT_reg_) both with and without further stimulation. Besides preTfr being suppressive, our results also indicate that preTfr cells exhibit noncanonical functions such as enhanced wound healing capacity in comparison to nT_regs_ (*31*, *42*). This function expands our understanding of Tfr cells beyond their conventional regulatory roles in the immune system and further distinguishes preTfr from nT_regs_.

We and others previously showed a reduction in total circulating Tfr in the blood of patients with severe COVID-19 (*15*, *18*). Here, we expand this by demonstrating that even at the CD45RA^+^ precursor state, the numbers are significantly reduced. This reduction suggests that Tfr cells are disrupted at early stages of their formation during severe disease, which could have implications for immune dysregulation and autoimmunity in these patients. We found a low percentage of preTfr in patients with COVID-19 who had a higher concentration of anti–IFN-γ autoantibodies, a critical cytokine for protection against COVID-19 (*43*). Alongside this, the CD45RB^lo^ activated atypical B cells that we previously described to be strongly associated with anti–SARS-CoV2 antibody responses (*23*) were increased in the same anti–IFN-γ autoantibody-positive donors and negatively correlated with the preTfr. We previously noted a relationship between total Tfr and atypical B cells during COVID-19 (*18*), but the recent discovery of preTfr and CD45RB^lo^ activated atypical B cells shows this in greater resolution. This would suggest that these cells may also be capable of the production of autoantibodies alongside anti–SARS-CoV antibodies. Furthermore, mortality of patients with COVID-19 was associated with a low frequency of preTfr but not cTfr. mRNA vaccination significantly, but subtly, increased the numbers of preTfr, which was further increased for each vaccination. Previous results have suggested that human cTfr are an indicator of ongoing GC reactions (*13*), and our data suggest that this may also be the case for preTfr, which we found to be positively correlated with vaccine-reactive subsets such as cTfh and CD45RB^lo^ activated atypical B cells during vaccination, although they lacked a clear relationship with neutralizing antibodies. It is particularly notable that while COVID-19 strongly reduced Tfr frequencies in the blood, mRNA vaccination increased them potentially, indicating a better controlled humoral response that does not generate autoantibodies.

Because of their stable and expandable nature, CD45RA^+^ T_regs_ have been used in a range of studies (*2*, *44*, *45*) as a source of cells for expansion and adoptive cell transfer to inhibit human autoimmune disease. Our results suggest that this population, previously believed to be homogeneous, is instead made of two separate groups of CXCR5-positive and CXCR5-negative cells. Since in most cases CXCR5 is not used to initially isolate these cells, this would imply that human clinical studies with CD45RA^+^ T_regs_ are using a mixed population of cells. CXCR5^+^ CD45RA^+^ preTfr appear to be a highly stable and suppressive population with unique characteristics that may be beneficial in this context. It may also be the case that specific isolation and expansion of this population may be well suited to further use as a clinical product aimed at targeting autoimmune diseases with notable components of autoantibody production such as SLE.

In summary, we have here shown that preTfr is a specific T_reg_ subset that acts as a precursor to cTfr, with important biological roles in severe inflammatory conditions. These findings may pave the way for therapeutic strategies for inflammatory conditions and autoimmune diseases.

## MATERIALS AND METHODS

### Human participants recruitment and sampling ICU cohort

Longitudinal collections of PBMC and plasma samples were obtained from cohorts including patients with moderate COVID-19 (non-ICU), patients with severe COVID-19 (ICU), patients with severe sepsis (ICU), and age-matched healthy controls as previously reported (*23*). Enrollment of patients with COVID-19 was based on clinical manifestations and polymerase chain reaction test results, while patients with sepsis were enrolled following sepsis-3 criteria. Healthy control subjects were recruited from The University of Osaka Hospital and Osaka General Medical Center. PBMC and plasma samples were also gathered from healthy donors during a vaccination time course with the Pfizer-BioNTech BNT162b2 SARS-CoV-2 vaccine, spanning from August 2020 to May 2021. We previously published a B cell–focused paper using the same dataset (*23*). This study complied with the principles outlined in the Declaration of Helsinki and received approval from the institutional review board of The University of Osaka Hospital [permit nos. 907 and 885 (The University of Osaka Critical Care Consortium Novel Omix Project; Occonomix Project)]. Informed consent was obtained from all participants. In vitro assays were performed using human PBMCs from healthy volunteer donor blood collected under ethical approval numbers 895 and 897 (The University of Osaka Research Ethics Committee) after informed consent or purchased from STEMCELL Technologies. Tonsil samples were approved by the Institutional Review Board of Nagoya City University Graduate School of Medical Sciences (protocol number: 60-00-1282).

### Peripheral blood and tonsil sample processing

PBMCs were extracted from fresh blood samples using Ficoll-Paque density gradient centrifugation. The isolated PBMCs were then resuspended at a concentration of 1 to 2 × 10^6^ cells/ml in Cellbanker 1 (Takara Bio). Tonsils from patients with hypertrophy and IgA nephropathy were cut into small sizes by scissors first and then mechanically processed using a gentleMACS dissociator (Miltenyi Biotec). The cells were filtered through a 70-μm cell strainer (Falcon) and then resuspended at a concentration of 1 to 5 × 10^7^ cells/ml in Cellbanker. These cells underwent controlled freezing at −80°C in a CoolCell device (Corning) before being stored in gas-phase liquid nitrogen.

### Mass cytometry antibody production

Indium-113, indium-115, and gadolinium-157 (Trace Sciences) and other lanthanide isotopes were linked to antibodies using X8 polymer MaxPar kits (Standard Biotools) and MCP9 polymer Maxpar kits for Cadmium isotopes (Standard Biotools). Platinum-labeled antibodies were conjugated with cisplatin following established methods (*46*). The conjugated antibodies were stored in a phosphate-buffered saline (PBS)–based antibody stabilizer or horseradish peroxidase protector stabilizer for cadmium labeling (Candor Biosciences). Each antibody was titrated with control PBMCs to determine the optimal staining concentrations. Antibody staining panels were either prepared fresh or in bulk and stored as aliquots at −80°C.

### CD45 barcoding and cell staining for mass cytometry

Mass cytometry staining was done as previously described (*23*). Briefly, samples were analyzed across 20 experimental runs. Frozen PBMC samples were thawed in a 37°C water bath for 2 min, transferred to a 15-ml tube, and mixed with 5 ml of prewarmed RPMI containing 10% fetal calf serum and Pierce Universal Nuclease for Cell Lysis (20 IU/ml). After washing, the samples were resuspended in 2 ml of CyFACS buffer, and live cells were counted using acridine orange/propidium iodide staining on a LUNA-FL fluorescence cell counter (Logos Biosystems). Up to 4 × 10^6^ viable cells per sample were labeled with a seven choose-three pattern of anti-CD45 barcodes (89Y, 113In, 115In, 194Pt, 195Pt, 196Pt, and 198Pt), allowing for up to 35 barcoded samples per experiment, including a spike-in control to monitor batch effects. Barcoding antibody aliquots were prepared in bulk and stored at −80°C.

Samples were incubated with CD45 barcodes and FC block in CyFACS buffer (PBS with 0.1% bovine serum albumin and 2 mM EDTA) for 30 min at room temperature, washed, resuspended in 700 μl of CyFACS buffer, pooled, and filtered through a 70-μm filter (Miltenyi Biotec) to remove debris. Cells were then separated into CD3^+^ and CD3^−^ fractions using a magnetic bead–based CD3-positive selection kit (STEMCELL Technologies). Each fraction was stained with surface and intracellular panels specific for T cells (CD4, Tfh, T_reg_, CD8, and γδT), non–T cells (monocytes, dendritic cells (DCs), B, and natural killer cells), and total lineage proportions. Premixed and frozen master mixes were used to reduce batch effects. Tonsil samples and healthy PBMCs from the in vitro plasmablast suppression assay was stained in a similar manner, except that the samples were not magnetically separated before staining and specialist antibody panels were used for each experiment (table S1).

### Mass cytometry data acquisition

Cells were washed and resuspended in Cell Acquisition Buffer (CAS, Standard Biotools). They were then diluted to 1.1 × 10^6^ cells/ml in CAS with 15% EQ Four Element Calibration Beads (Standard Biotools) and filtered through a 35-μm filter before collection. The cells were run at 200 to 500 cells/s on a Helios mass cytometer (Standard Biotools) or CyTOF XT. FCS files were normalized to EQ beads, concatenated using Standard Biotools software, and exported for analysis.

### Mass cytometry data analysis

FCS files were uploaded to Cytobank software (Beckman Coulter), gated for DNA positivity, EQ bead negativity, Zr live/dead stain below the threshold, and normal ion cloud Gaussian parameters. The files were then manually debarcoded using CD45 barcodes and exported as FCS files. The data were imported into R (v.4.3.0) using flowCore (v.2.12.2) and batch corrected with cyCombine (v.0.2.15), with spike-in samples confirming the accuracy of batch correction. Missing markers were imputed using cyCombine functions. Postbatch correction, samples were converted to single-cell experiment format and analyzed with CATALYST (v.1.24.0). The data were compensated using a compensation matrix derived from single-metal–labeled compensation beads, clustered using FlowSOM, and subclustered for detailed cell type annotation.

### ForceAtlas2 visualization

To visualize T_regs_ using the ForceAtlas2 algorithm, 5000 cells per cluster were generated to generate a k-nearest neighbors (KNN) edge matrix in Vortex software. The resulting XML file was imported into Gephi (v.0.10.1) for layout, with the ForceAtlas2 algorithm settings configured as follows: tolerance = 1, approximate repulsion = 1.2, scaling = 1, gravity = 4, and dissuade hubs ON.

### Detection of anti-cytokine autoantibodies by multiplex bead array

The levels of anti-cytokine autoantibodies IL-1α, IL-6, IL-10, IL-22, IL-17A, and IFN-γ in plasma samples were determined using the MILLIPLEX Human Cytokine Autoantibody Expanded IgG kit (Millipore) according to the manufacturer’s instructions. The assay was run on a BioPlex-200 machine (Bio-Rad). Median fluorescent intensity (MFI) measurements greater than 2 SDs above the average MFI for healthy controls were considered positive.

### Detection of anti–IFN-α by ELISA

The Human anti–IFN-α ELISA Kit (Invitrogen) was run according to the manufacturer’s directions using 1:5 diluted plasma samples from the patients or controls.

### Quantification of SARS-CoV-2 neutralizing antibodies

The SARS-CoV-2 neutralizing antibody ELISA Kit (Invitrogen) was run according to the manufacturer’s directions using 1:50 diluted plasma samples from the mRNA vaccine recipients. The post-first time point (2 to 4 days following the vaccination) was not included in analysis as it was expected to be too early for the development of neutralizing antibodies.

### Bulk RNA-seq of naïve T_reg_ and Tfr and effector T_reg_ and Tfr cells

#### Cell isolation and sorting

nT_reg_ and preTfr cells were isolated from human PBMCs using FACS. nT_regs_ were sorted as CD4^+^CD25^+^CD127^−^ CD45RA^+^CD45RO^−^CXCR5^−^ cells, while preTfr cells were sorted as CD4^+^CD25^+^CD127^−^CD45RA^+^CD45RO^−^CXCR5^+^cells. eT_regs_ were sorted as CD4^+^CD25^+^CD127^−^CD45RA^−^CD45RO^+^CXCR5^−^, and cTfr cells were CD4^+^CD25^+^CD127^−^CD45RA^−^CD45RO^+^CXCR5^+^.

#### RNA extraction, library preparation, and RNA-seq

Total RNA was extracted from cells with an miRNeasy Micro kit (QIAGEN) according to the manufacturer’s protocol. Full-length cDNA was generated using a SMART-Seq HT Kit (Takara Bio) according to the manufacturer’s instructions. An Illumina library was prepared using the Nextera XT DNA Library Prep Kit (Illumina) according to SMARTer kit instructions. Sequencing was performed on a NovaSeq 6000 sequencer (Illumina) in the 101–base paired-end mode.

### Bulk RNA-seq data analysis

Next-generation sequencing read quality was assessed with FastQC (v. 0.11.9) and MultiQC (v. 1.12). Adaptor sequences were trimmed, and low-quality reads were removed using Trimmomatic (v. 0.39). All sequencing reads aligning (HiSAT2, v. 2.2.1) to annotated ribosomal RNA genes were discarded. High-quality and ribosomal RNA-depleted sequencing reads were aligned to the human genome (GRCh38) using HiSAT2. Using sorted bam files (Samtools v. 1.15), the number of aligned reads was counted (featurecount in subread package v. 2.0.1). After normalization (trimmed mean of *M* values), a differential gene expression analysis (edgeR v. 4.0.3) was performed in R (v. 4.3.0). Significant differentially expressed (DE) genes were distinguished by a false discovery rate (FDR) under 0.05. Gene ontology analysis was performed in R (v. 4.3.0) with clusterProfiler (v. 4.10.0) and org.Hs.eg.db (v. 3.18.0). All scripts are available on Github: https://github.com/jonasns/nTfr.

### Cell isolation and flow cytometry

CD4^+^ T cells were isolated from PBMCs using the CD4^+^ T Cell Isolation Kit (Miltenyi Biotec) following the manufacturer’s instructions. The cells were isolated using AutoMACS Pro Separator (Miltenyi Biotec). The cells were then processed using the “Depletes” program, and the negative fraction containing CD4^+^ fraction was used for cell sorting of CD4^+^ T cell subsets, and the CD4^−^ fraction was used for sorting naïve B cells. T cells were stained with anti-CD45RA BV711 (BioLegend), anti-CD4 APC-Cy7 (BD Pharmingen), anti-CD127 AF647 (BD Pharmingen), anti-CXCR5 BV421 (BD Biosciences), anti-CD45RO fluorescein isothiocyanate (FITC; BD Pharmingen), anti-CD25 phycoerythrin (PE; BD Pharmingen), anti-CD19 V500 (Bioscience), anti-FOXP3 PE (eBioscience), anti-Helios FITC (BioLegend), and anti-BCL6 Alexa Fluor 647 (BD Pharmingen). B cells were stained with anti-CD19 V500 (Bioscience), anti-CD38 PE (BD Pharmingen), anti-CD27 PE-Cy7 (BioLegend), and anti-IgD Alexa Fluor 488 (BioLegend). For cell viability staining, the Live/Dead Fixable Aqua Dead Cell Stain Kit (Life Technologies) was used. Carboxyfluorescein diacetate succinimidyl ester (CFSE; Life Technologies) was used to assess cell proliferation. Cell sorting was performed using Aria III (BD Biosciences). Flow cytometry analysis was performed with an LSRFortessa (BD Biosciences) and further analyzed with FlowJo v10 software.

### Cell culture and functional assays

For in vitro cell expansion, 4 × 10^3^ CD45RA^+^CD45RO^−^CXCR5^−^ nT_reg_ or 3 × 10^3^ CD45RA^+^CD45RO^−^CXCR5^+^ preTfr cells were activated by human T_reg_ expander beads (Miltenyi Biotec) and cytokine cocktails of IL-2 (500 IU/ml; Shionogi), activin A (100 ng/ml; PeproTech), and IL-12 (1 ng/ml; PeproTech). After day 8, beads were removed by magnetic separation, and cells were resupplemented with cytokine cocktails. After day 14, cells were restimulated by beads. The media was supplemented with 2% human AB blood type serum (Gemcell, H49K007), GlutaMAX (10%), and a mixture of glutamine and penicillin-streptomycin (10%). An OpTmizer T Cell Expansion (Thermo Fisher Scientific) media was added with OpTmizer T Cell Expansion supplement. For T cell suppression assays, 1 × 10^4^ CD4^+^CD25^−^CD127^+^CXCR5^−^ conventional T cells were plated with responder cells nT_reg_ or preTfr cells diluted in 1:1, 1:3, and 1:7. Responder cells were labeled with CFSE (Invitrogen) and were stimulated with IL-2 (10 IU/ml) T_reg_ inspector beads (Miltenyi Biotec) according to the manufacturer’s instructions or gamma irradiations of APCs. After 5 days, cells were harvested, and responder cells were analyzed for CFSE dilution by flow cytometry. For in vitro B cell suppression assays, 5 × 10^4^ CD4^+^CD25^−^CD127^+^CXCR5^+^ Tfh cells were plated with nT_reg_ or preTfr cells in a 1:1 ratio in the presence of 5 × 10^4^ CD19^+^ IgD^+^ naïve B cells. Cells were cultured with Staphylococcal enterotoxin B (SEB) (100 ng/ml) (Sigma-Aldrich). After 6 days, the frequency of CD38^+^ IgD^−^ plasmablast was determined by flow cytometry.

Cultures were performed in U-shaped 96-well plates in RPMI 1640 (Nakalai) supplemented with 10% fetal bovine serum (Gibco), 1% penicillin-streptomycin, and glutamine (Gibco) in 37°C, 5% CO_2_ incubator conditions.

### Generation of SN for suppression and in vitro wound healing assay

Freshly isolated human nT_reg_ or preTfr cells (1 × 10^5^) were sorted and cultured for 3 days in TexMACS medium (Miltenyi Biotec) supplemented with human T cell TransAct (Miltenyi Biotec: anti-CD3– and anti-CD28–based stimulation) and either IL-2 (500 U/ml) alone or IL-2 (500 U/ml) combined with IL-12, IL-21, IL-23, and transforming growth factor–β (each at 50 ng/ml; PeproTech), referred to as the TPC. The culture conditions followed a previously described protocol (*31*). On the third day, fresh cytokines were added to the cultures, and the cells were maintained for an additional 2 to 3 days. After a total of 5 to 6 days, cells were washed twice to remove residual cytokines and resuspended in fresh medium containing IL-2 and TransAct for a 20-hour incubation, the culture SN was collected, and cells were analyzed for flow cytometry. SNs were used for wound healing and in vitro plasmablast suppression assay.

### Total PBMC plasmablast suppression assay

A total of 1 × 10^5^ PBMCs per well were plated in 96-well U-bottom plates and treated by recombinant IL-1RA (40 ng/ml; BioLegend), and the SN from TPC assay of nT_reg_ and preTfr cells was diluted in 1:8 and blocking antibody with anti–IL-1RA monoclonal antibody (20 μg/ml; JK1RA-1, BioLegend). After 2 hours of incubation, cells were stimulated with anti-CD3 (1 μg/ml; BioLegend), IL-2 (5 IU/ml), and IL-1β (2 ng/ml; PeproTech), incubated for 5 days, and analyzed by CyTOF assay.

### In vitro wound healing assay with HaCaT cells

The human keratinocyte cell line HaCaT (RRID: CVCL_0038) was grown in TexMACS medium supplemented with 1% penicillin/streptomycin. HaCaT cells were seeded in a culture-insert (ibidi culture-insert 2 well, ibidi GmbH) according to the manufacturer’s instructions. After allowing the cells to attach overnight, we removed the culture-insert and washed the cells with PBS to remove nonadherent cells. We then provided fresh medium containing preTfr and nT_reg_ SNs diluted at 1:15 and cultured for 48 hours and incubated at 37°C. Microscope images were taken using KEYENCE, BZ-X710 at ×4 magnification. The open area was quantified using ImageJ (1.53) wand selection tool with four-connected mode and a tolerance of 2 (*47*).

### Figure production

Figures were assembled in Adobe Illustrator (v.27.3-28.0).
