# Supplementary material for: Human precursor T follicular regulatory cells are primed for differentiation into mature Tfr and disrupted during severe infections
Source: Sci Adv. 2025 Sep 26;11(39):eadv6939. doi: 10.1126/sciadv.adv6939 (PMC12467056; doi:10.1126/sciadv.adv6939)
Supplement: Supplementary file 1 — Figs. S1 to S12 Table S1 [file sciadv.adv6939_sm.pdf]

Science Advances
AAAS

Supplementary Materials for

Human precursor T follicular regulatory cells are primed for differentiation into mature Tfr and disrupted during severe infections

Janyerkye Tulyeu *et al.*

Corresponding author: James B. Wing, jbwing@ifrec.osaka-u.ac.jp

Sci. Adv. **11**, eadv6939 (2025)
DOI: 10.1126/sciadv.adv6939

This PDF file includes:

Figs. S1 to S12
Table S1

Supplementary Materials

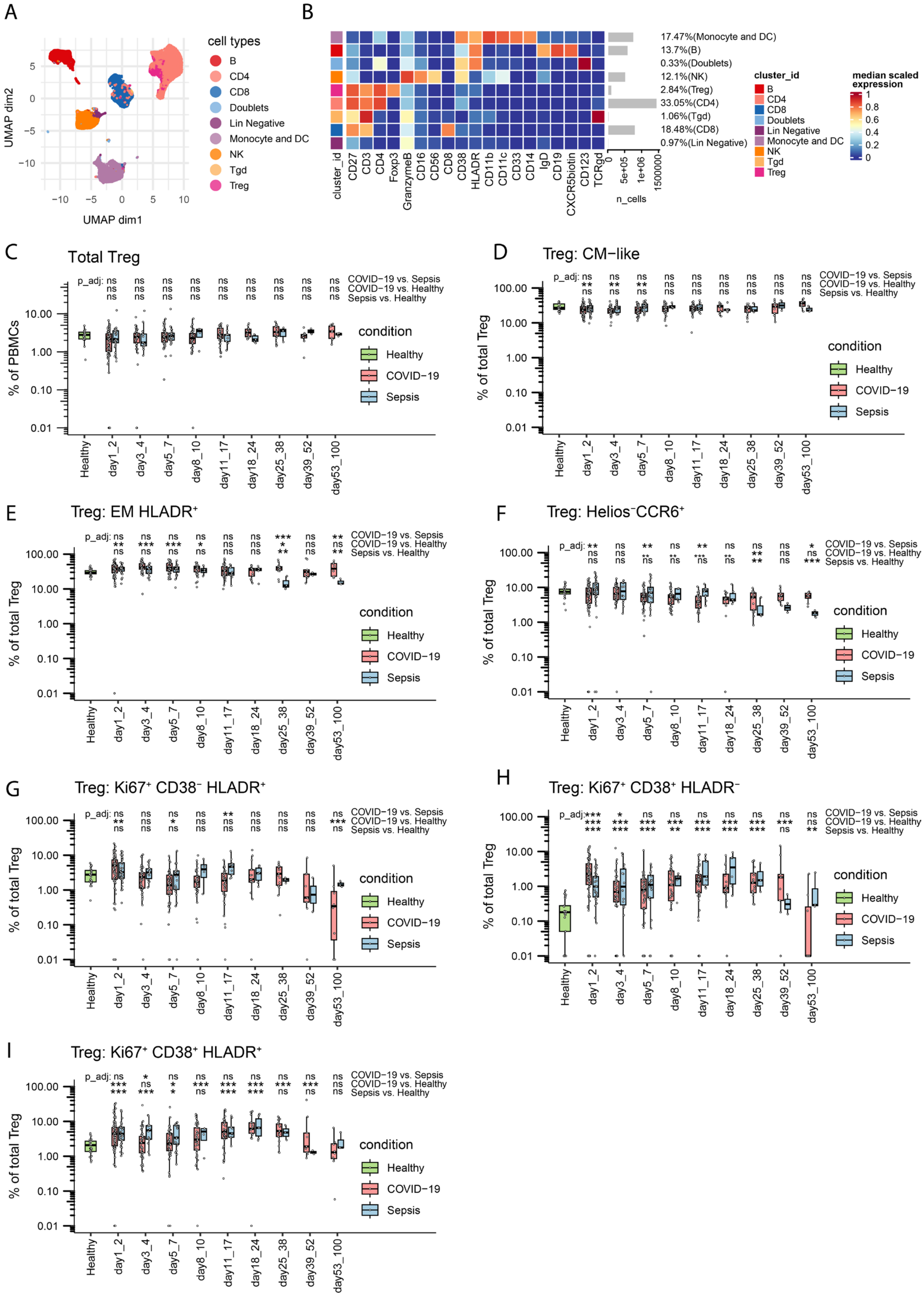

Fig. S1: Proportion of Treg cell subsets in COVID-19 and Sepsis ICU patients over time.
(**A**) UMAP visualization of main immune cell lineages in total PBMCs. (**B**) Heatmap illustrating the median scaled expression of various cell type markers across total PBMC FlowSOM clusters. (**C-I**) Proportion of total Treg and Treg cell subsets in COVID-19 and Sepsis ICU patients compared to age-matched healthy controls over time. Statistical significance (FDR-adjusted p-values from edgeR) is indicated with $*p < 0.05$, $**p < 0.01$, $***p < 0.001$. Healthy, n = 25. Day 1-2, COVID-19 n =85, sepsis n = 34; day 3-4, COVID-19 n =43, sepsis n = 14; day 5-7, COVID-19 n =52, sepsis n = 20; day 8-10, COVID-19 n =38, sepsis n = 4; day 11-17, COVID-19 n =38, sepsis n = 9; day 18-24, COVID-19 n =16, sepsis n = 6; day 25-38, COVID-19 n =17, sepsis n = 3; day 39-52, COVID-19 n =9, sepsis n = 2; day 53-100, COVID-19 n =8, sepsis n = 3. For all boxplots, hinges correspond to the first and third quartiles, and whiskers correspond to the 1.5-times interquartile range.

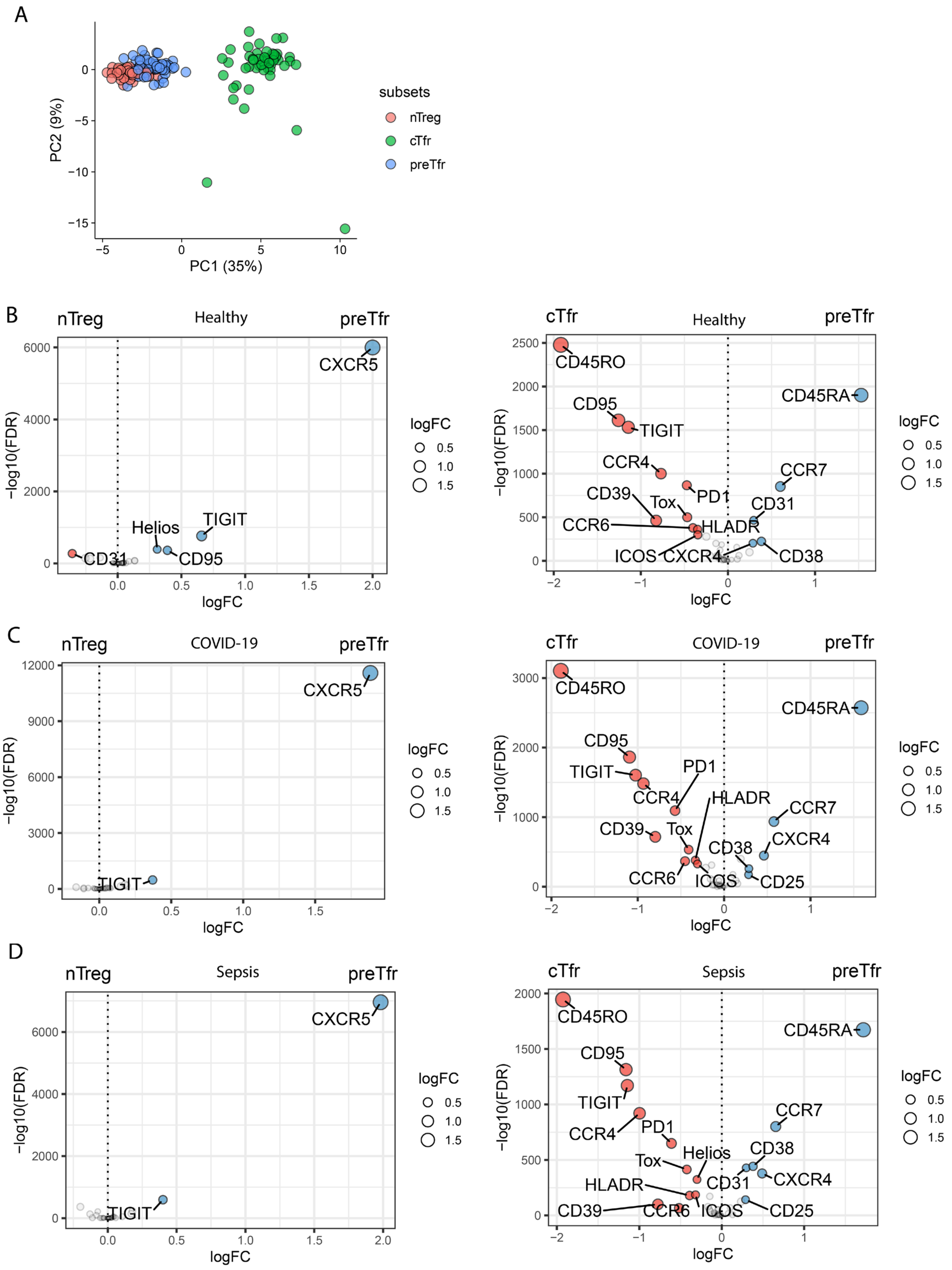
A
PC2 (9%)
PC1 (35%)
subsets
nTreg
cTfr
preTfr
B
nTreg
Healthy
preTfr
CXCR5
TIGIT
Helios
CD31
CD95
−log10(FDR)
logFC
cTfr
Healthy
preTfr
CD45RO
CD45RA
CD95
TIGIT
CCR4
PD1
CCR7
CD39
Tox
CD31
CCR6
HLADR
ICOS
CXCR4
CD38
C
nTreg
COVID-19
preTfr
CXCR5
TIGIT
cTfr
COVID-19
preTfr
CD45RO
CD45RA
CD95
TIGIT
PD1
CCR4
HLADR
CCR7
CD39
Tox
CXCR4
CD38
CCR6
ICOS
CD25
D
nTreg
Sepsis
preTfr
CXCR5
TIGIT
cTfr
Sepsis
preTfr
CD45RO
CD45RA
CD95
TIGIT
CCR7
CCR4
PD1
CD38
Helios
Tox
CD31
CXCR4
HLADR
CD39
CCR6
ICOS
CD25

Fig. S2: Comparison of marker expression between Treg subsets in COVID-19, sepsis and healthy controls.

(**A**) PCA of Treg subsets in healthy controls. Each dot represents one donor (n=53).

(**B-D**) Volcano plots showing the results of differential expression analysis comparing markers between Treg subsets in healthy controls (b, n=53), COVID-19 (c, n=97), and sepsis (d, n=38). The compared Treg subsets are indicated above the graph. Statistics: scran/findmarkers with Wilcoxon rank sum test corrected for donor differences.

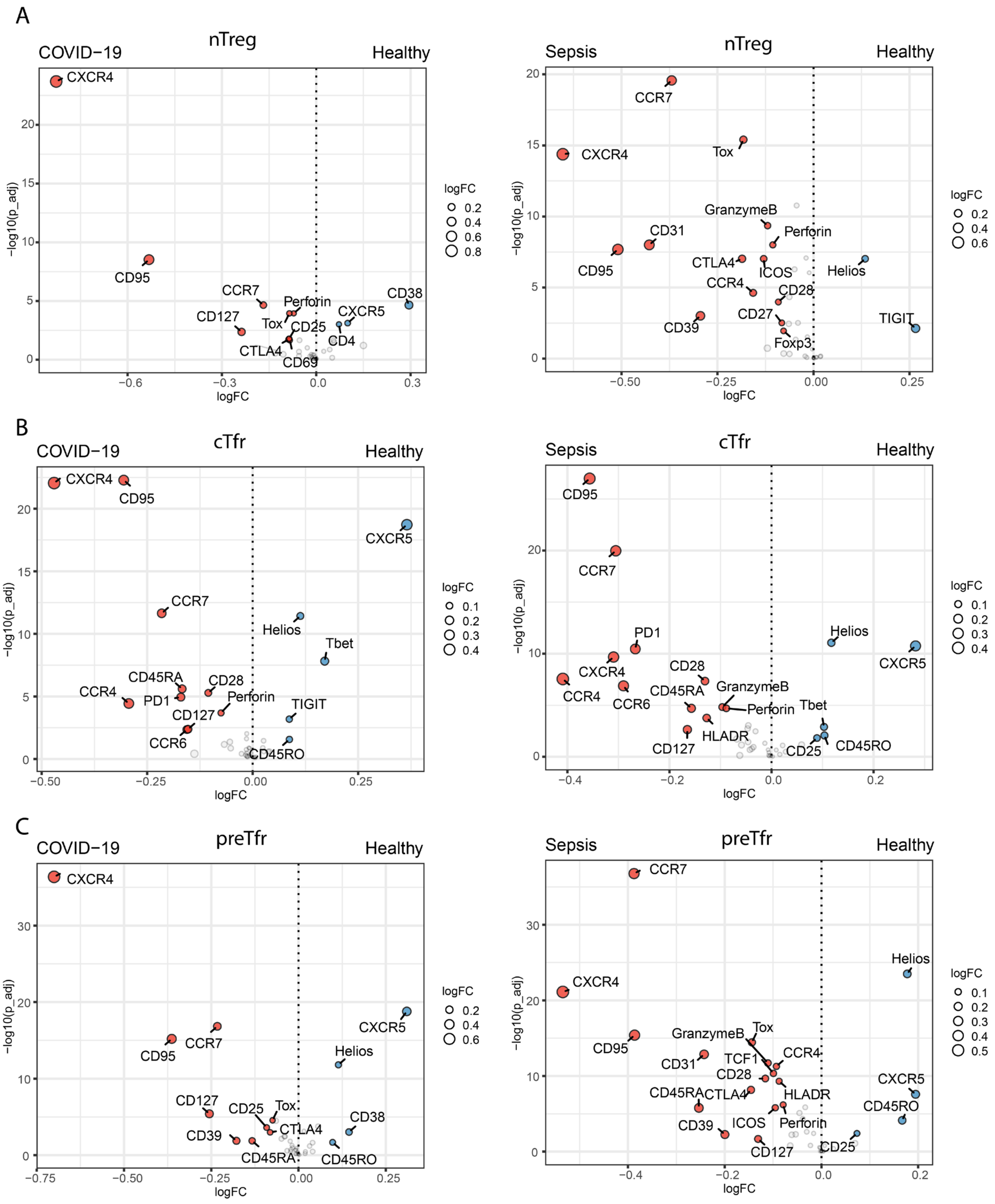
A
COVID−19
nTreg
Healthy
CXCR4
CD95
CCR7
Perforin
CD127
Tox
CD25
CXCR5
CD38
CD4
CTLA4
CD69
−log10(p_adj)
logFC
logFC
0.2
0.4
0.6
0.8
Sepsis
nTreg
Healthy
CCR7
Tox
CXCR4
GranzymeB
Perforin
CD31
CD95
CTLA4
ICOS
Helios
CCR4
CD28
CD27
CD39
TIGIT
Foxp3
−log10(p_adj)
logFC
logFC
0.2
0.4
0.6
B
COVID−19
cTfr
Healthy
CXCR4
CD95
CXCR5
CCR7
Helios
Tbet
CD45RA
CD28
CCR4
PD1
Perforin
TIGIT
CD127
CCR6
CD45RO
−log10(p_adj)
logFC
logFC
0.1
0.2
0.3
0.4
Sepsis
cTfr
Healthy
CD95
CCR7
PD1
Helios
CXCR5
CXCR4
CD28
CCR4
GranzymeB
CD45RA
CCR6
Perforin
Tbet
HLADR
CD127
CD25
CD45RO
−log10(p_adj)
logFC
logFC
0.1
0.2
0.3
0.4
C
COVID−19
preTfr
Healthy
CXCR4
CXCR5
CD95
CCR7
Helios
CD127
CD25
Tox
CTLA4
CD38
CD39
CD45RA
CD45RO
−log10(p_adj)
logFC
logFC
0.2
0.4
0.6
Sepsis
preTfr
Healthy
CCR7
Helios
CXCR4
GranzymeB
Tox
CD95
TCF1
CCR4
CD31
CD28
CXCR5
CD45RA
CTLA4
HLADR
CD45RO
CD39
ICOS
Perforin
CD127
CD25
−log10(p_adj)
logFC
logFC
0.1
0.2
0.3
0.4
0.5

Fig. S3: Comparison of marker expression for each Treg subset between COVID-19, sepsis and healthy controls.

(**A-C**) Volcano plots showing the results of differential expression analysis comparing healthy controls (n=25), COVID-19 (n=97), and sepsis (n=38) for markers on each Treg subsets. The compared Treg subset is indicated above the graph. Statistics: diffcyt/limma, significant results are indicated in blue or red (p-adj < 0.05).

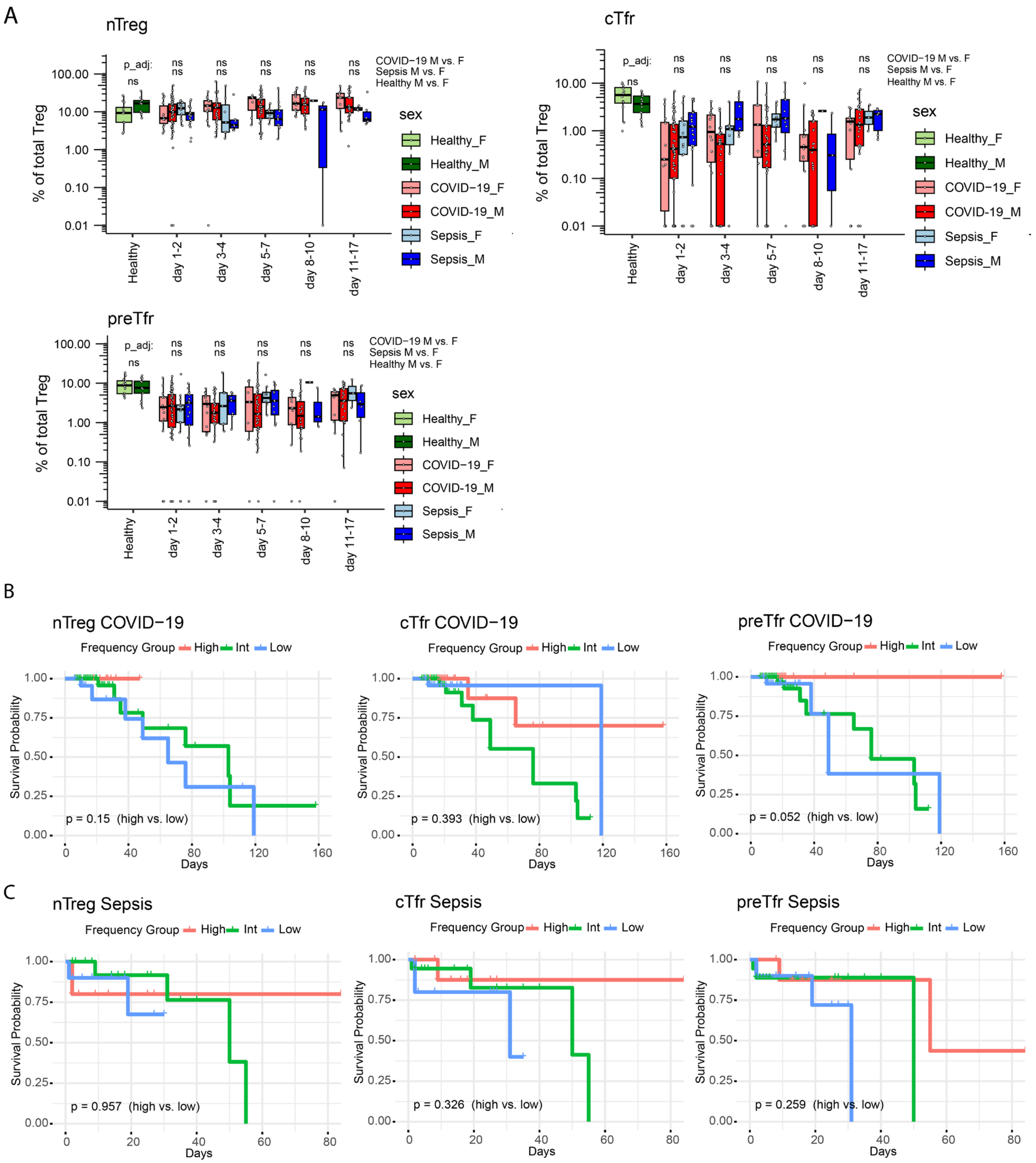

Fig. S4: Sex- and mortality relationship with Treg subset frequencies in COVID-19 and Sepsis.

(**A**) Box plots of the percentage of nTreg, cTfr and preTfr in COVID-19, Sepsis and healthy controls divided by gender, F = female, M = male. Statistics: FDR-adjusted p-values from edgeR. Healthy, n = 25. Day 1-2, COVID-19 n =85, sepsis n = 34; day 3-4, COVID-19 n =43, sepsis n =

14; day 5-7, COVID-19 n =52, sepsis n = 20; day 8-10, COVID-19 n =38, sepsis n = 4; day 11-17, COVID-19 n =38, sepsis n = 9. Boxplot hinges correspond to the first and third quartiles, and whiskers correspond to the 1.5-times interquartile range. ns: non-significant.

(**B-C**) Kaplan-Meier plot of survival versus days in ICU for b) COVID-19 and c) Sepsis patients. Patients were divided based on the upper and lower quartile of the average frequency of Treg subsets. COVID-19 survivors n=82, Sepsis survivors n=30; COVID-19 non-survivors n=15, Sepsis non-survivors n=8. Statistics: Log-rank test comparing only higher versus lower quartile frequency.

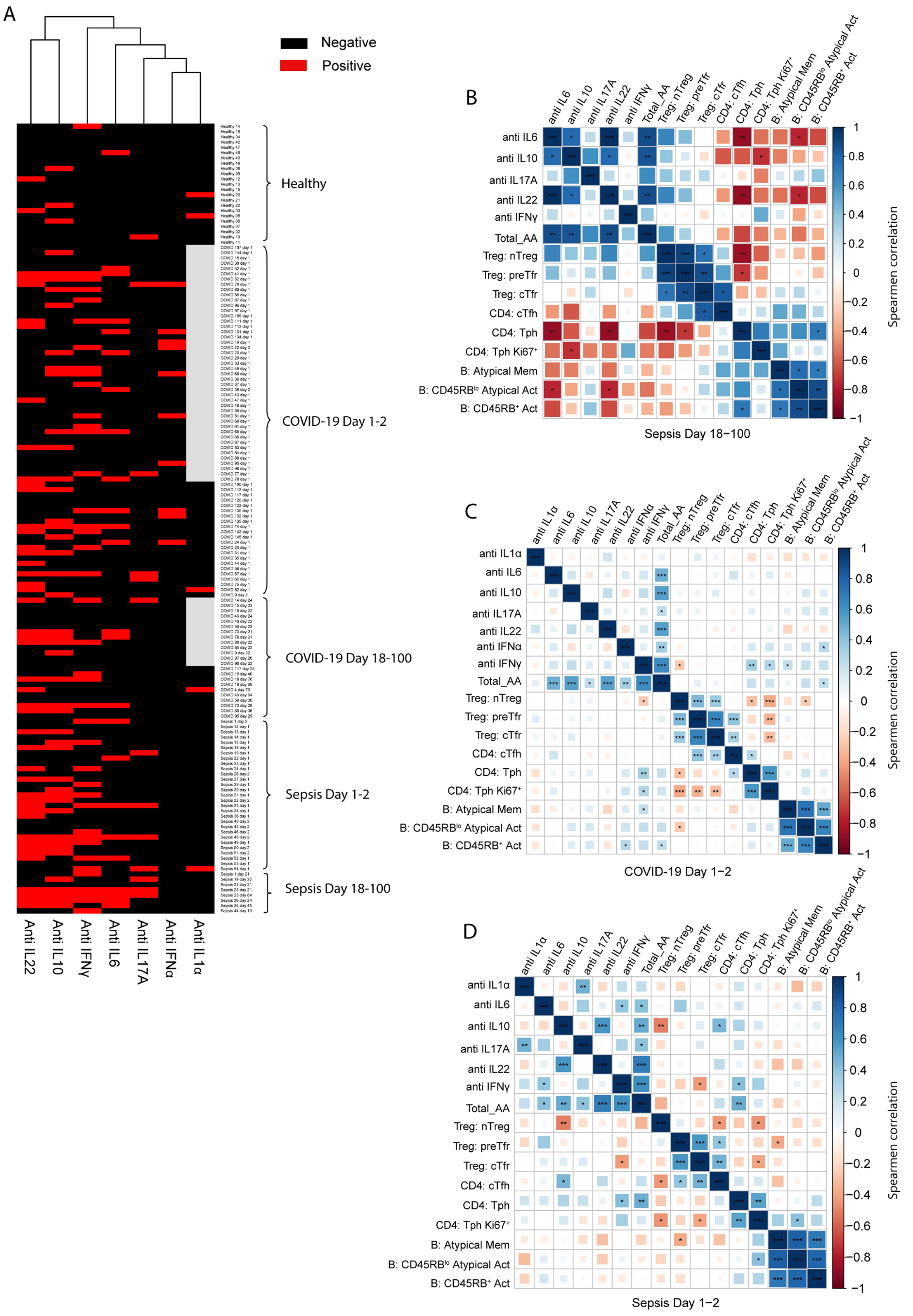
A
Negative
Positive
Healthy
COVID-19 Day 1-2
COVID-19 Day 18-100
Sepsis Day 1-2
Sepsis Day 18-100
Anti IL22
Anti IL10
Anti IFNγ
Anti IL6
Anti IL17A
Anti IFNα
Anti IL1α
B
anti IL6
anti IL10
anti IL17A
anti IL22
anti IFNγ
Total_AA
Treg: nTreg
Treg: preTfr
Treg: cTfr
CD4: cTfh
CD4: Tph
CD4: Tph Ki67⁺
B: Atypical Mem
B: CD45RBlo Atypical Act
B: CD45RB⁺ Act
Sepsis Day 18−100
Spearmen correlation
C
anti IL1α
anti IL6
anti IL10
anti IL17A
anti IL22
anti IFNα
anti IFNγ
Total_AA
Treg: nTreg
Treg: preTfr
Treg: cTfr
CD4: cTfh
CD4: Tph
CD4: Tph Ki67⁺
B: Atypical Mem
B: CD45RBlo Atypical Act
B: CD45RB⁺ Act
COVID-19 Day 1−2
Spearmen correlation
D
anti IL1α
anti IL6
anti IL10
anti IL17A
anti IL22
anti IFNγ
Total_AA
Treg: nTreg
Treg: preTfr
Treg: cTfr
CD4: cTfh
CD4: Tph
CD4: Tph Ki67⁺
B: Atypical Mem
B: CD45RBlo Atypical Act
B: CD45RB⁺ Act
Sepsis Day 1−2
Spearmen correlation

Fig. S5: Anti-cytokine Autoantibody detection in COVID-19, Sepsis, and healthy controls. (**A**) Heatmap showing the detection of anti-cytokine autoantibodies in individual COVID-19 (n=90), Sepsis (n=37) patients, and healthy controls (n=23). Colors represent autoantibodies with MFI measurement greater than 2 standard deviations (SD) above the average MFI for healthy controls (red = positive) or less than 2 SD below (black = negative). The gray color indicates the specific patients were not tested for this anti-cytokine autoantibody. (**B-D**) Spearman rank correlation matrix of indicated autoantibodies and Treg subsets in late and early COVID-19 and Sepsis patients (COVID-19 n=89, Sepsis n=36). Significance *$p<0.05$, ** $p<0.01$, *** $p<0.001$ by two-sided Spearman's rank correlation.

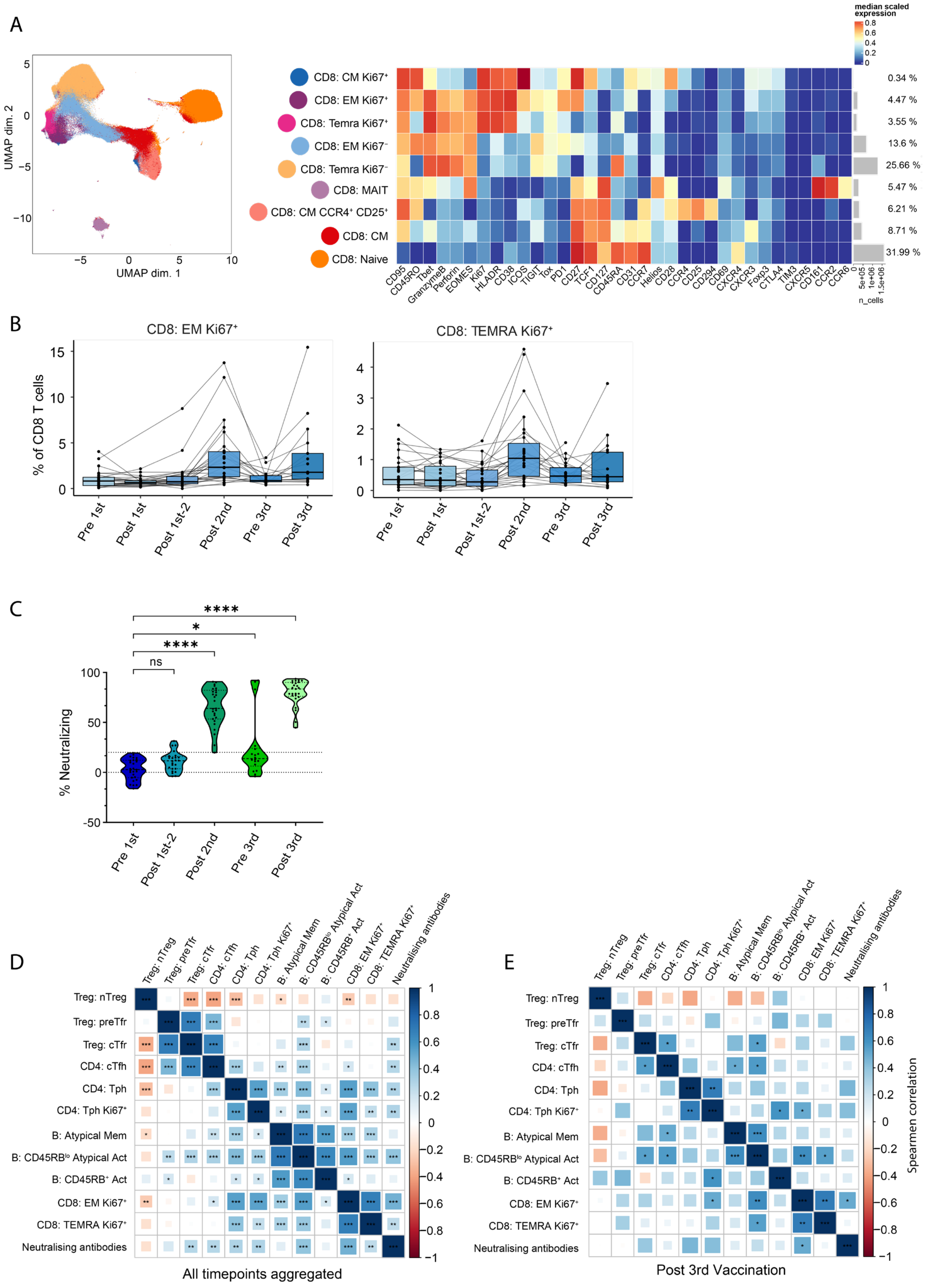
A
UMAP dim. 2
UMAP dim. 1
median scaled expression
CD8: CM Ki67⁺
CD8: EM Ki67⁺
CD8: Temra Ki67⁺
CD8: EM Ki67⁻
CD8: Temra Ki67⁻
CD8: MAIT
CD8: CM CCR4⁺ CD25⁺
CD8: CM
CD8: Naive
0.34 %
4.47 %
3.55 %
13.6 %
25.66 %
5.47 %
6.21 %
8.71 %
31.99 %
CD95 CD45RO Tbet GranzymeB Perforin EOMES Ki67 HLADR CD38 ICOS TIGIT Tox PD1 CD27 TCF1 CD127 CD45RA CD31 CCR7 Helios CD28 CCR4 CD25 CD294 CD69 CXCR4 CXCR3 Foxp3 CTLA4 TIM3 CXCR5 CD161 CCR2 CCR6
n_cells
B
CD8: EM Ki67⁺
CD8: TEMRA Ki67⁺
% of CD8 T cells
Pre 1st
Post 1st
Post 1st-2
Post 2nd
Pre 3rd
Post 3rd
C
% Neutralizing
ns
*
****
Pre 1st
Post 1st-2
Post 2nd
Pre 3rd
Post 3rd
D
E
Treg: nTreg
Treg: preTfr
Treg: cTfr
CD4: cTfh
CD4: Tph
CD4: Tph Ki67⁺
B: Atypical Mem
B: CD45RBˡᵒ Atypical Act
B: CD45RB⁺ Act
CD8: EM Ki67⁺
CD8: TEMRA Ki67⁺
Neutralising antibodies
All timepoints aggregated
Post 3rd Vaccination
Spearmen correlation

Fig. S6: Neutralizing antibodies and CD8 T-cells during vaccination.

(A) UMAP visualization of $CD8^+$ T cell lineages in total PBMCs and heatmap illustrating the median scaled expression of various cell type markers across $CD8^+$ T-cell FlowSOM clusters. **(B)** Proportions of selected $CD8^+$ T cell clusters within the SARS-CoV-2 mRNA vaccination cohort over time. **(C)** Anti-SARS-CoV2 Neutralizing antibody levels within plasma samples from the SARS-CoV-2 mRNA vaccination cohort over time. Dotted line at 20% indicates threshold for being considered positive. n = 27, 24, 27, 18 and 25 per indicated time point. Significance *p<0.05, **** p<0.0001 by Kruskal Wallis with Dunn's post test. Levels determined by ELISA. **(D-E)** Spearman rank correlation matrix of neutralizing antibodies, Tregs and vaccine responsive B-cell, $CD4^+$ T and $CD8^+$ T-cell populations at indicated timepoints. Significance *p<0.05, ** p<0.01, *** p<0.001 by two-sided Spearman's rank correlation (n = 123-156 in D, and n = 17-28 in E).

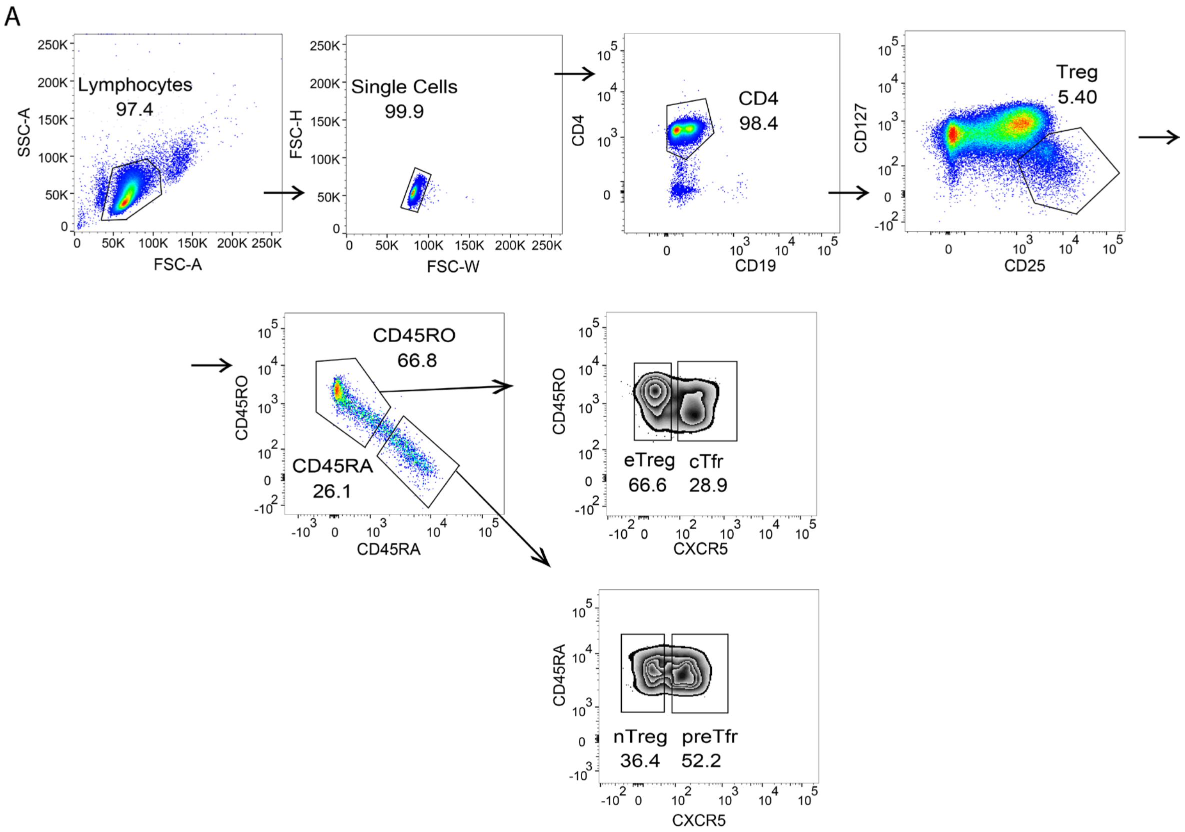

Fig. S7: Gating strategy for preTfr FACS sorting

Gating strategy to identify and isolate naïve Treg and Tfr cell subsets from $CD4^+$ T cells by FACS.

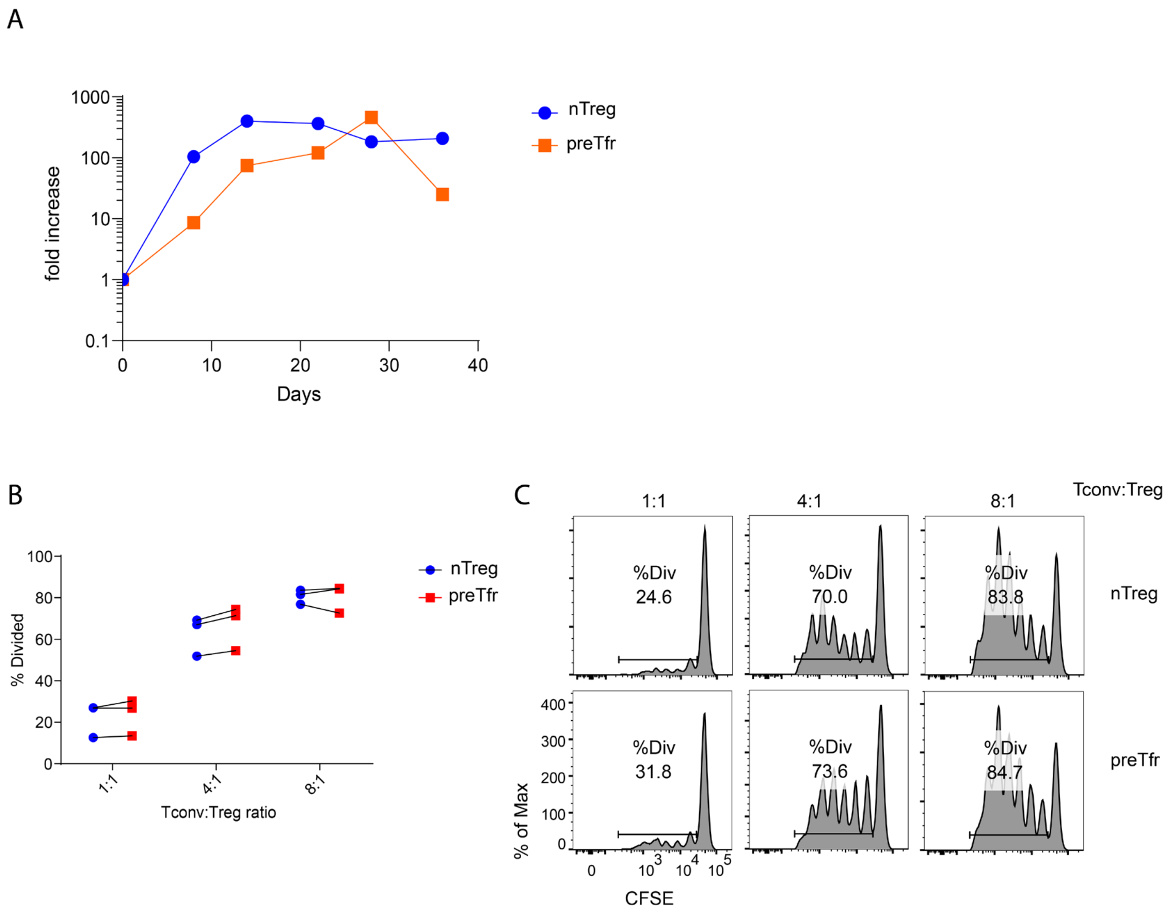

Fig. S8: preTfr expansion and Treg suppression assays.

(**A**) Line graph depicting the expansion of nTreg and preTfr cells over time (days in culture, n=1). (**B**) Percentage of proliferating Tconv cells in the presence of varying concentrations of Tregs and antigen-presenting cells (n=3). (**C**) Histograms comparing the percentage of proliferating Tconv across different conditions.

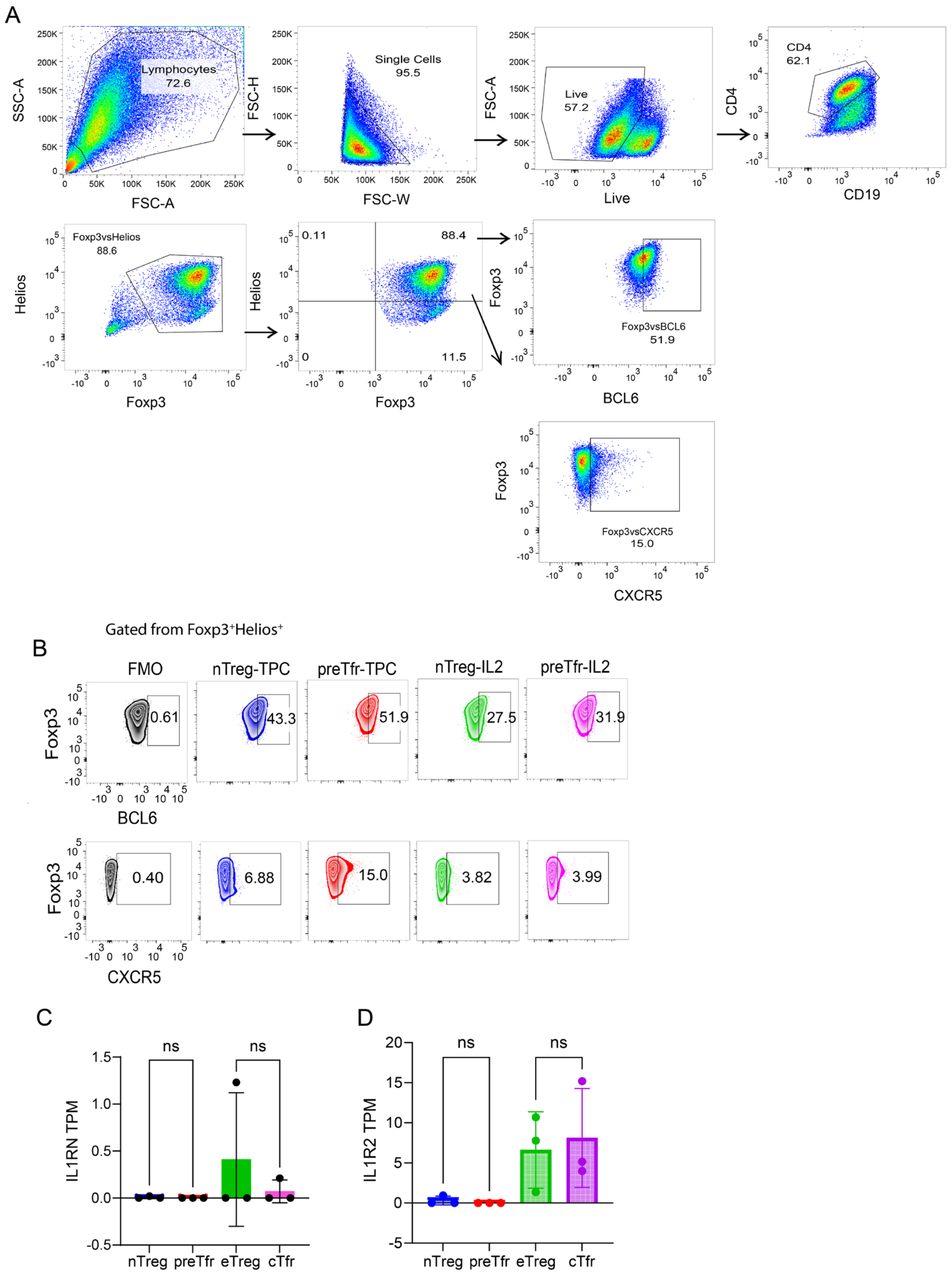

Fig. S9: Gating strategy for FACS phenotyping cells in TPC assay. (**A, B**) Gating strategy for expression of represented markers. (**C, D**) mRNA expression levels of IL1RN and IL1R2. Statistics: edgeR with paired design, n = 3 per condition from fresh sorted Treg subsets.

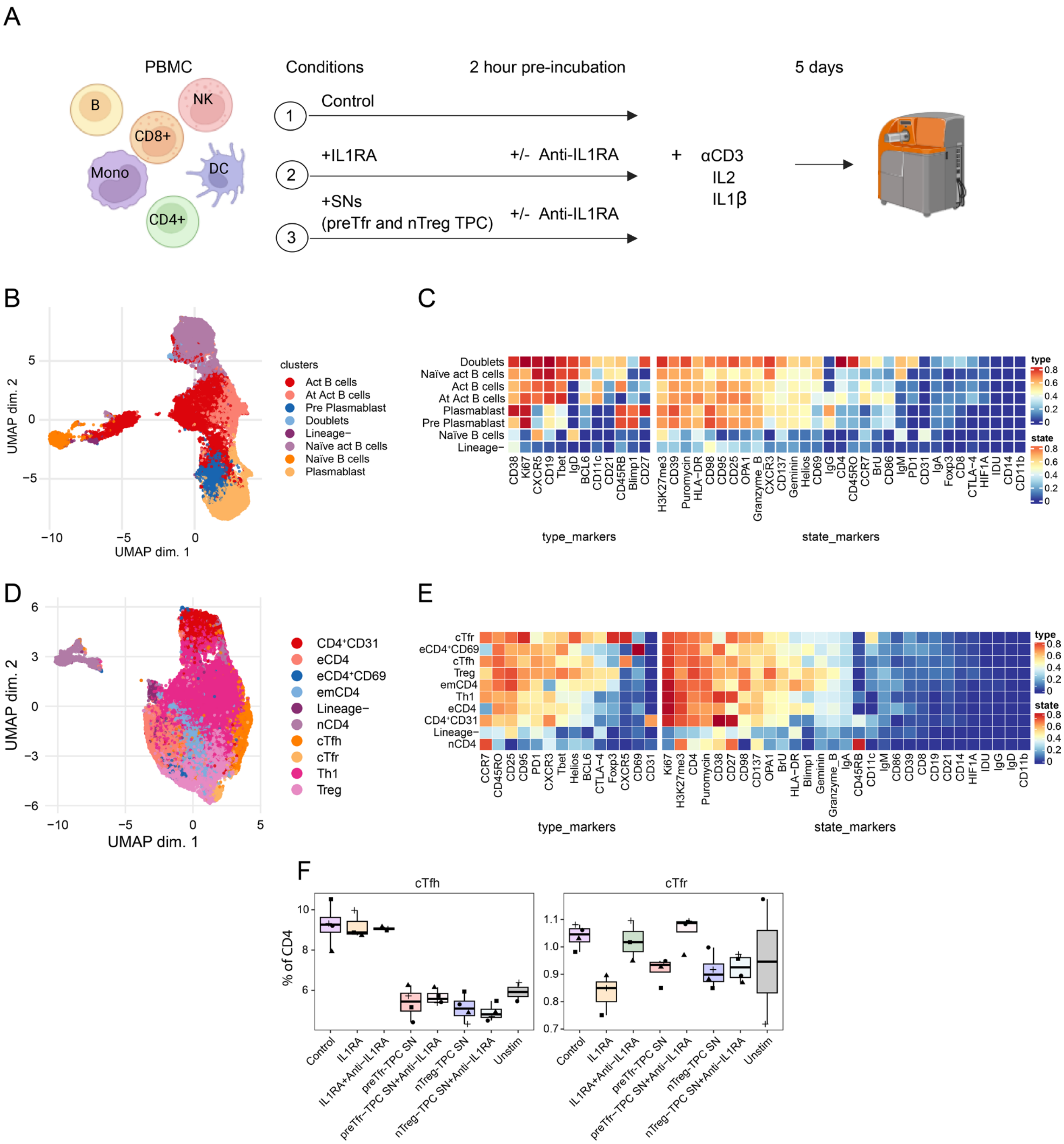

A
PBMC
B
NK
CD8+
Mono
DC
CD4+
Conditions
2 hour pre-incubation
5 days
1
Control
2
+IL1RA
+/- Anti-IL1RA
+
αCD3
IL2
IL1β
3
+SNs
(preTfr and nTreg TPC)
+/- Anti-IL1RA
B
UMAP dim. 2
UMAP dim. 1
clusters
Act B cells
At Act B cells
Pre Plasmablast
Doublets
Lineage−
Naïve act B cells
Naïve B cells
Plasmablast
C
Doublets
Naïve act B cells
Act B cells
At Act B cells
Plasmablast
Pre Plasmablast
Naïve B cells
Lineage−
CD38 Ki67 CXCR5 CD19 Tbet IgD BCL6 CD11c CD21 CD45RB Blimp1 CD27
H3K27me3 CD39 Puromycin HLA−DR CD98 CD95 CD25 OPA1 Granzyme_B CXCR3 CD137 Geminin Helios CD69 IgG CD4 CD45RO CCR7 BrU CD86 IgM PD1 CD31 IgA Foxp3 CD8 CTLA−4 HIF1A IDU CD14 CD11b
type_markers
state_markers
type
state
D
UMAP dim. 2
UMAP dim. 1
CD4⁺CD31
eCD4
eCD4⁺CD69
emCD4
Lineage−
nCD4
cTfh
cTfr
Th1
Treg
E
cTfr
eCD4⁺CD69
cTfh
Treg
emCD4
Th1
eCD4
CD4⁺CD31
Lineage−
nCD4
CCR7 CD45RO CD25 CD95 PD1 CXCR3 Tbet Helios BCL6 CTLA−4 Foxp3 CXCR5 CD69 CD31
Ki67 H3K27me3 CD4 Puromycin CD38 CD27 CD98 CD137 OPA1 BrU HLA−DR Blimp1 Geminin Granzyme_B IgA CD45RB CD11c IgM CD86 CD39 CD8 CD19 CD21 CD14 HIF1A IDU IgG IgD CD11b
type_markers
state_markers
type
state
F
cTfh
cTfr
% of CD4
Control
IL1RA
IL1RA+Anti-IL1RA
preTfr-TPC SN
preTfr-TPC SN+Anti-IL1RA
nTreg-TPC SN
nTreg-TPC SN+Anti-IL1RA
Unstim

Fig. S10: Marker expression and subset frequency in B cell and CD4 T-cell subsets from plasmablast suppression assay.

(**A**) Schematic representation of the *in vitro* total PBMC Plasmablast suppression assay. Created in BioRender. Wing, J. (2025) https://BioRender.com/fdl8gzw. (**B-C**) UMAP and Heatmap of median scaled expression of markers used for clustering of B-cells (type-markers) or not (state-markers). (**D-E**) UMAP and Heatmap of median scaled expression of markers used for clustering of CD4 T-cells (type-markers) or not (state-markers). (**F**) Boxplots of selected CD4 T-cell subsets from the plasmablast suppression assay in total PBMCs treated with TPC culture SNs.

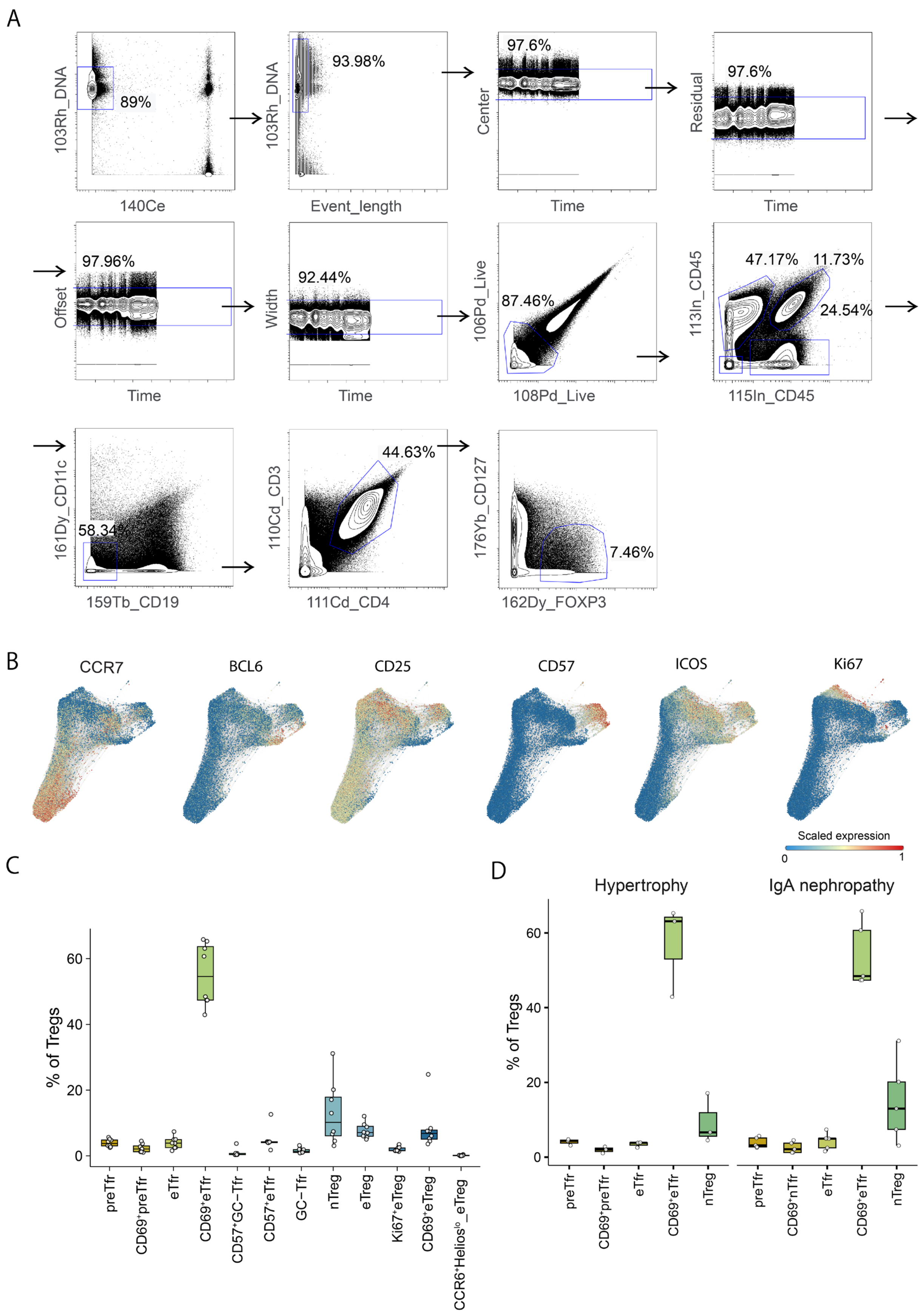
A
103Rh_DNA
140Ce
89%
93.98%
Event_length
97.6%
Center
Time
97.6%
Residual
Time
97.96%
Offset
Time
92.44%
Width
Time
87.46%
106Pd_Live
108Pd_Live
47.17%
11.73%
24.54%
113In_CD45
115In_CD45
161Dy_CD11c
159Tb_CD19
58.34%
44.63%
110Cd_CD3
111Cd_CD4
176Yb_CD127
162Dy_FOXP3
7.46%
B
CCR7
BCL6
CD25
CD57
ICOS
Ki67
Scaled expression
0
1
C
% of Tregs
preTfr
CD69⁺preTfr
eTfr
CD69⁺eTfr
CD57⁺GC−Tfr
CD57⁺eTfr
GC−Tfr
nTreg
eTreg
Ki67⁺eTreg
CD69⁺eTreg
CCR6⁺Helios^lo_eTreg
D
Hypertrophy
IgA nephropathy
% of Tregs
preTfr
CD69⁺preTfr
eTfr
CD69⁺eTfr
nTreg
preTfr
CD69⁺nTfr
eTfr
CD69⁺eTfr
nTreg

Fig. S11: Mass cytometry analysis of regulatory T cell populations in human tonsils.

(**A**) Representative gating strategy of Treg cells from CyTOF assay. (**B**) Expression of specific protein markers shown on ForceAtlas2 plot. (**C**) The frequency of all Treg clusters in total Treg of human tonsil. (**D**) The frequency of selected Treg clusters in human tonsils grouped by patient group.

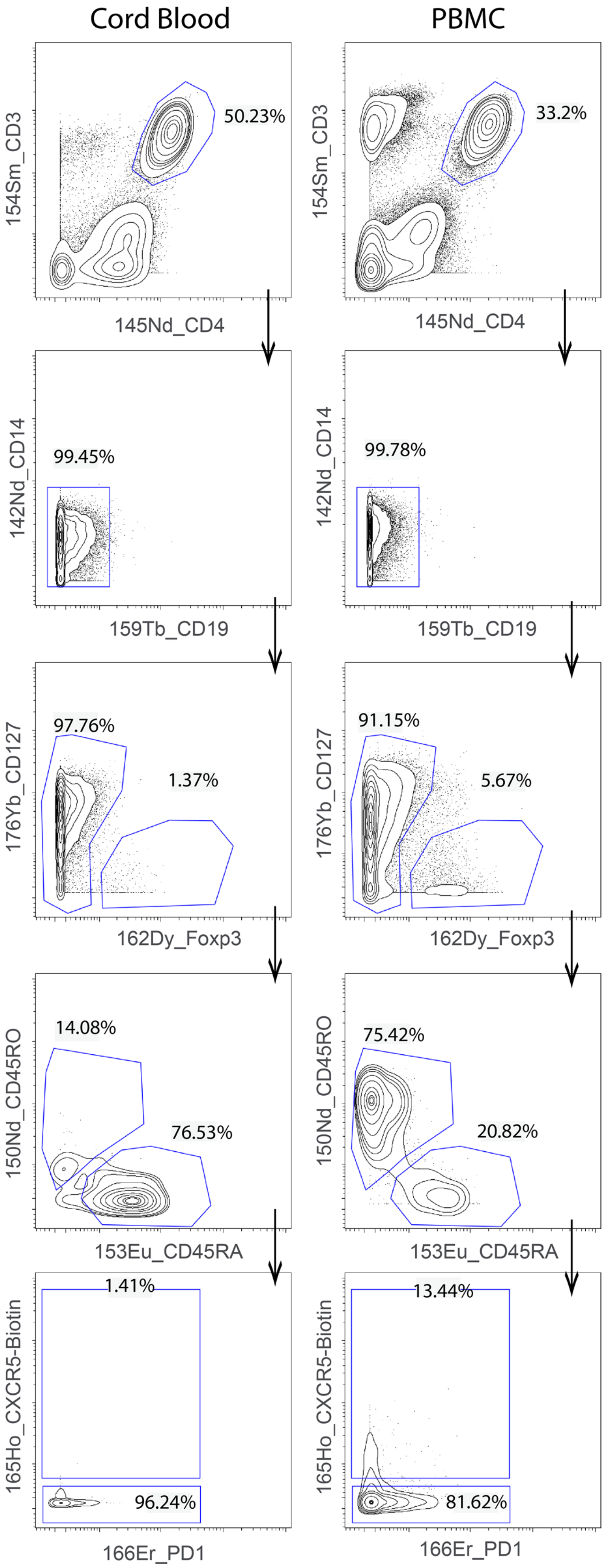

Cord Blood
PBMC
154Sm_CD3
145Nd_CD4
50.23%
33.2%
142Nd_CD14
159Tb_CD19
99.45%
99.78%
176Yb_CD127
162Dy_Foxp3
97.76%
1.37%
91.15%
5.67%
150Nd_CD45RO
153Eu_CD45RA
14.08%
76.53%
75.42%
20.82%
165Ho_CXCR5-Biotin
166Er_PD1
1.41%
96.24%
13.44%
81.62%

Fig. S12: Gating strategy for cord blood and PBMCs assayed by mass cytometry.

Representative gating strategy of Treg subsets from cord blood and PBMC. Cells were gated as $CD3^{+}CD4^{+}CD19^{-}CD14^{-}Foxp3^{+}CD45RA^{+}$ cells before gating on CXCR5.

Supplementary table 1. Mass Cytometry Panels

**Barcode and Prestain**					
**Label**	**Target**	**Manufacturer**	**Clone**	**Cat Number**	**Titration**
	Fc block	Biolegend	-	422302	50
154Sm	anti-CXCR3	Biolegend	G025H7	353733	100
155Gd	anti-CCR7	Biolegend	G043H7	353202	100
127I	IdU	Standard Biotools	-	201127	100
NA	Puromycin	Sigma	-	P8833	50
NA	BrU	Sigma	-	850187	20
Biotin	anti-CXCR5	BD	RF8B2	552118	25
89Y	anti-CD45	Standard Biotools	HI30	3089003B	75
113In	anti-CD45	Biolegend	HI30	304002	100
115In	anti-CD45	Biolegend	HI30	304002	100
194Pt	anti-CD45	Biolegend	HI30	304002	100
195Pt	anti-CD45	Biolegend	HI30	304002	100
196Pt	anti-CD45	Biolegend	HI30	304002	100
**Surface Stain**					
**Label**	**Target**	**Manufacturer**	**Clone**	**Cat Number**	**Titration**
106Cd	anti-CD45RO	Biolegend	UCHL1	304202	50
110Cd	anti-CD21	Biolegend	Bu32	354902	100
111Cd	anti-CD4	Biolegend	RPA-T4	300502	200
112Cd	anti-CD8	Biolegend	RPA-T8	301002	50
114Cd	anti-CD14	Biolegend	M5E2	301810	100
116Cd	anti-CD19	Biolegend	HIB19	302202	100
116Cd	anti-CD28	Biolegend	CD28.2	302902	50
142Nd	anti-CD137	Biolegend	4-1BB	309802	50

147Sm	anti-CD86	Biolegend	IT2.2	305402	50
151Eu	anti-IgD	Biolegend	IA6-2	348202	100
152Sm	anti-CD69	Biolegend	N50	310902	100
158Gd	anti-CD27	Standard Biotools	LG.3A10	3150017B	200
160Gd	anti-CD39	Standard Biotools	A1	3160004B	100
164Dy	anti-CD95	Biolegend	DX2	305631	100
165Ho	anti-Biotin (for CXCR5)	Standard Biotools	1D4C5	3165012B	50
166Er	anti-PD1	Biolegend	EH12.2H7	329941	100
167Er	anti-CD31	Biolegend	WM59	303127	200
169Tm	anti-CD25	Standard Biotools	2A3	3169003B	200
171Yb	anti-CD98	BD	UM7F8	556074	200
172Yb	anti-CD38	Standard Biotools	HIT2	3172007B	100
173Yb	anti-CD11c	Biolegend	S-HCL-3	371502	100
174Yb	anti-HLA-DR	Biolegend	L243	307602	200
176Yb	anti-CD45RB	Biolegend	MEM-55	310202	200
209Bi	anti-CD11b	Standard Biotools	ICRF44	3209003B	100
198Pt	Cell-ID™ Cisplatin-198Pt (Live Dead stain)	Standard Biotools	-	201198	-
**Intracellular Stain**					
**Label**	**Target**	**Manufacturer**	**Clone**	**Cat Number**	**Titration**
103Rh	Cell-ID Intercalator-Rh (DNA stain)	Standard Biotools	-	201103A	
140Ce	anti-H3K27me3	Active motive	323	61017	400
141Pr	Anti-puromycin	Sigma			400
143Nd	anti-IgM	Biolegend	MHM-88	314502	200
146Nd	anti-Helios	Biolegend	22F6	137202	100
149Sm	anti-BLIMP1	R&D Systems	MAB36081	646702	100
150Nd	anti-Granzyme B	NOBUSBIO	CLB-GB11	NBP1-50071	100
153Eu	anti-IgG	Biolegend	M1310G05	410701	200
156Gd	anti-BrdU (for BrU)	BD	3D4	555627	50
157Gd	anti-OPA1	AbCam	EPR11057(B)	ab240143	100
159Tb	anti-Geminin	Proteintech	Polyclonal	10802-1-AP	50
161Dy	anti-T-bet	Biolegend	4B10	644802	100

162Dy	anti-Foxp3	eBioscience	236A/E7	14-4777-82	100
163Dy	anti-BCL6	Abcam	K112-91	ab307682	50
168Er	anti-Ki67	Standard Biotools	Ki67	3168001B	100
170Er	anti-CTLA-4	Standard Biotools	14D3	3170005B	50
175Lu	anti-HIF1A	Thermo	16H4L13	700505	200
